# Prevalence and Risk Factors of Infection with High Risk Human Papilloma Viruses among HIV-Positive Women with Clinical Manifestations of Tuberculosis in a Middle-Income Country

**DOI:** 10.3390/biomedicines9060683

**Published:** 2021-06-16

**Authors:** Maria Isaguliants, Marina Nosik, Anastasia Karlsen, Natalia Petrakova, Marina Enaeva, Natalia Lebedeva, Daria Podchufarova, Vita Laga, Konstantin Gromov, Anatoly Nazarov, Sona Chowdhury, Mikhail Sinitsyn, Alexander Sobkin, Natalya Chistyakova, Svetlana Aleshina, Alexei Grabarnik, Joel M. Palefsky

**Affiliations:** 1 Department of Microbiology, Tumor and Cell Biology, Karolinska Institutet, 17177 Stockholm, Sweden; 2 Institute of Microbiology and Virology, Riga Stradins University, LV-1007 Riga, Latvia; 3 N.F. Gamaleya National Research Center for Epidemiology and Microbiology, 123098 Moscow, Russia; karlsen12@gmail.com (A.K.); nvpetrakova@hotmail.com (N.P.); vitaq5laga@gmail.com (V.L.); konstantinhiv@bk.ru (K.G.); 4 I.I. Mechnikov Institute of Vaccine and Sera, 105064 Moscow, Russia; mnossik@yandex.ru; 5 Medical Academy for Continuous Professional Education, 125993 Moscow, Russia; 6 Moscow Clinical Scientific Center Named after A.S. Loginov, 111123 Moscow, Russia; doc.enaeva.marina@gmail.com; 7 Moscow Regional Center for Prevention and Control of AIDS and Infectious Diseases, 129110 Moscow, Russia; lebedevanatalya@rocketmail.com (N.L.); biochemic83@yandex.ru (D.P.); 8 West Medica, 129075 Moscow, Russia; a.nazarov@westmedica.com; 9 Department of Medicine, University of California San Francisco, San Francisco, CA 94143, USA; Sona.Chowdhury@ucsf.edu (S.C.); Joel.Palefsky@ucsf.edu (J.M.P.); 10 Moscow Scientific and Clinical Center for TB Control, 107076 Moscow, Russia; msinitsyn@mail.ru (M.S.); stefany01@mail.ru (S.A.); a.grabarnik@mail.ru (A.G.); 11 G.A. Zaharyan Moscow Tuberculosis Clinic, Department for Treatment of TB Patients with HIV Infection, 125466 Moscow, Russia; alexandr@sobkin.net (A.S.); thistyakova@yandex.ru (N.C.)

**Keywords:** women living with HIV-1, AIDS, sociodemographic characteristics, *M. tuberculosis*, clinical TB manifestations, ART and TB treatment, prevalence of high risk HPVs, sequence of HPV 16 E6 and E7, therapeutic HPV vaccine

## Abstract

Women living with HIV-1 are at high risk of infection with human papillomavirus of high carcinogenic risk (HR HPVs). *M. tuberculosis* (TB) promotes HPV infection and increases the risk to develop HPV-associated cancer. Our knowledge of persisting HR HPVs genotypes, and of the factors promoting HR HPV infection in people living with HIV-1 with clinical TB manifestations is sparse. Here, we analyzed 58 women living with HIV-1 with clinical TB manifestations (WLWH with TB) followed up in specialized centers in Russia, a middle income country endemic for HIV-1 and TB, for the presence in cervical smears of DNA of twelve HR HPV genotypes. DNA encoding HPV16 E5, E6/E7 was sequenced. Sociodemographic data of patients was collected by questionnaire. All women were at C2-C3 stages of HIV-infection (by CDC). The majority were over 30 years old, had secondary education, were unemployed, had sexual partners, experienced 2–3 pregnancies and at least one abortion, and were smokers. The most prevalent was HPV16 detected in the cervical smears of 38% of study participants. Altogether 34.5% of study participants were positive for HR HPV types other than HPV16; however, but none of these types was seen in more than 7% of tested samples. Altogether, 20.7% of study participants were positive for several HR HPV types. Infections with HPVs other than HPV16 were common among WLWH with generalized TB receiving combined ART/TB-therapy, and associated with their ability to work, indirectly reflecting both their health and lifestyle. The overall prevalence of HR HPVs was associated with sexual activity of women reflected by the number of pregnancies, and of HPV 16, with young age; none was associated to CD4+-counts, route of HIV-infection, duration of life with HIV, forms of TB-infection, or duration of ART, characterizing the immune status. Thus, WLWH with TB—especially young—were predisposed to infection with HPV16, advancing it as a basis for a therapeutic HPV vaccine. Phylogenetic analysis of HPV16 E5, E6/E7 DNA revealed no common ancestry; sequences were similar to those of the European and American HPV16 strains, indicating that HPV vaccine for WLWH could be the same as HPV16 vaccines developed for the general population. Sociodemographic and health correlates of HR HPV prevalence in WLWH deserve further analysis to develop criteria/recommendations for prophylactic catch-up and therapeutic HPV vaccination of this highly susceptible and vulnerable population group.

## 1. Introduction

Up to 4.5% of new cases of cancer registered worldwide—including cervical, anogenital, and head and neck cancers—are associated with human papillomavirus (HPV) infection [1]. Cervical cancer (CC) accounts for 83% of these cases. According to the World Health Organization (WHO), cervical cancer caused by high oncogenic risk HPV (HR HPV) is the fourth most common cancer in women in the world [2]. In 2018, 311,000 women died from cervical cancer. WHO predicts that by 2045 cervical cancer will cause 491,000 deaths/year of women worldwide, and this figure will only increase in the future [3]. According to the experts, today the average incidence of cervical cancer worldwide is 13.1 cases per 100,000 women [4]. Globally the rate of cervix cancer is the highest in the age group 50–54 years [4].

The highest incidence of cervical cancer is in the countries of South, East, and West Africa, where the standardized rates are 43.1, 40.1, and 29.6 cases per 100,000 population, respectively [2,4]. In the high-income level countries, the total burden of cervical cancer is four times lower and is 4.1–8.7 cases per 100,000. In Russia, cervical cancer accounts for 5.2% of cancer cases among women [5]. Over the last five years, the number of patients with CC has increased by 2.4% and currently constitutes 22 cases per 100,000 women. The largest proportion of mortality from CC in Russia (23.7%) is in the age group of 30–39 years. This is a very alarming fact as the global average age at death from cervical cancer is reported to be 59 years (ranging from 45 years in Vanuatu to 76 years in Martinique) [4].

Studies have shown that 71% of cervical cancers are caused by persistent HR HPV infection, primarily genotypes 16 and 18 [6,7]. HPV 16 and 18 together with HR HPV types 31, 33, 45, 52, and 58 are responsible for over 90% of cervical cancer cases [1,8]. Women living with HIV-1 (WLWH) are significantly more likely to develop cervical cancer [9,10,11,12,13,14,15]. In WLWH, many more cases of CC are caused by HR HPV infection than in HIV-1 negative population (48% to 73% compared to 28%, respectively) [10,11,12,13,14]. Furthermore, the risk of reinfection increases 4–5 times [16,17] and mortality from cervical cancer is doubled [18,19]. The risk of infection with HR HPV types and subsequent development of cervical abnormalities in WLWH correlates with HIV-1 viral load and low CD4 cell counts [20,21] attributing HPV-related CC in WLWH to HIV-1 induced immune suppression.

The disparity in CC incidence between WLWH and HIV-1 negative women is specifically high in the low- and middle-income countries. In Africa, HIV-infected women are six times more likely to develop CC than HIV-negative women. The prevailing hypothesis is that HIV-mediated immune dysfunction leads to longer and more severe cervical HPV infections increasing the likelihood of oncogenic transformations which further develop into cervical cancer [22]. This scenario is specifically actual in the absence of regular access to health control, lack of knowledge on CC and CC screening options, and no access to these options, no access to antiretroviral therapy (ART) or late initiation of ART, as well as poor adherence to treatment [22,23]. The reasons for the disparity in CC incidence between WLWH and HIV-1 negative women in middle-income countries, not subjected or less subjected to the above limitations, are much less clear.

People living with HIV are 20 to 30 times more likely to be infected with *M. tuberculosis* and develop active tuberculosis (TB) with over 95% of TB deaths occurring in low- and middle-income countries [24]. Over the last 15 years, the economic development of middle-income countries—especially the BRICS, including Russia—has led to an increase in health spending, strengthening of primary care services, and significant decrease in TB prevalence. According to the report of the Russian Ministry of Health, the number of TB cases in Russia decreased between 2009 and 2019 by >50% (from 133,229 people in 2000 to 60,531 people in 2019) [25]. Still, the TB rates in Russia remain very high, especially among vulnerable groups in the large cities [26], and WLWH represent such vulnerable group. According to our study of 377 patients done in tuberculosis clinics in Moscow between 2015–2016, 56.8% of HIV-positive individuals had TB [27].

Infection with *M. tuberculosis* is a co-factor promoting HPV infection. TB causes chronic inflammation of organs and tissues and increases the risk of developing malignant neoplasms, including those in the gynecological tract [28,29]. Up to 67% of TB cases presenting with inflammation of the cervix are associated with infection by HR HPVs [30,31]. A study done in 8798 women found that in women who reported a TB diagnosis, or inflammation of the cervix, or both TB and inflammation of the cervix, the risk of persistent HR HPV infection increased by 75%, 22%, and 113%, respectively [30]. Furthermore, recent evidence has shown that infection with *M. tuberculosis* (MTB), specifically drug-resistant strains, induces host metabolic reprogramming, so called “immunometabolism”, modulating the capacity of the body to produce innate and through this, adaptive immune responses [32,33].

Today, 20.1 million women live with HIV-1 and 3.2 million with tuberculosis worldwide [34]. Infections grossly overlap jointly contributing to the immune suppression and dysfunction of the immune system. Analysis of the data on HR HPV infections in WLWH and in women with tuberculosis indicates that co-infection with HIV-1 and *M. tuberculosis* may strongly predispose to the infection with HR HPVs and consequent development of cervical abnormalities culminating in cervical cancer (more than single HIV-1 and MTB infections). Today, despite the pandemic character of HIV-1 and TB infections and the four-decades-long history of their co-circulation, the actual information on HR HPVs prevalence among women living with HIV-1 with clinical TB manifestations is sparse [31], and for middle- or high-income countries ironically unavailable, despite significant resources spent on healthcare and escalating TB spread linked to rising immigration. Knowledge on the characteristics of HR HPV variants persisting in women living with HIV-1 with tuberculosis, and demographic, clinical, social, and behavioral factors associated with acquisition of HR HPVs in the middle income countries is totally missing (we found no systematic studies published in the international peer-reviewed journals). Collection and analysis of these data is needed to help develop and personalize prevention and treatment programs for decreasing morbidity and mortality from cervical cancer in this highly vulnerable group of population.

## 2. Materials and Methods

### 2.1. Ethical Aspects

The study was approved by the Biomedical Ethics Committee of the N.F. Gamaleja National Research Center for Epidemiology and Microbiology, Moscow, Russia (protocol no. 11 of 3 July 2017). All participants gave written informed consent to take part in the study. According to the General Data Protection Regulation (GDPR) requirements, all participants were de-identified and anonymized by assigning them unique codes expressed as an identifier, based on the name of the individual and year of birth. Anonymization was done by medical doctors responsible for the patients. Patient samples were collected by qualified medical personnel. All materials, data, and study results were stored in an anonymized form.

### 2.2. Study Group of Women Living with HIV-1 with Clinical Manifestations of Tuberculosis (TB)

Study group enrolled 58 women living with HIV-1 with clinical manifestations of TB (WLWH with TB) (Supplementary Table S1), both naïve to treatment, i.e.,not receiving antiretroviral therapy (ART) or anti-tuberculosis therapy (TB therapy), and receiving ART and TB therapy in inpatient care center for treatment of TB patients with HIV infection at the G.A. Zaharyan Moscow Tuberculosis Clinic, Moscow, Russia, and at the Clinic No. 2 of the Moscow Scientific and Clinical Center for TB Control, Moscow, Russia. Clinical characteristics of patients were extracted from the medical records of patients in lines with the study protocol. Patients were diagnosed as HIV-seropositive by ELISA and by Western blot. The CD4+ T cell counting was performed by two-color flow cytometry using phycoerythrin labelled anti-CD4 antibodies (FACSort, Becton Dickinson, Franklin Lakes, NJ. USA). Demographic, sociological data in the study group were collected using a questionnaire.

### 2.3. Clinical Characteristics

All women in the study group were at C2–C3 stage of HIV-infection (CDC): C2—53.4% and C3—43.1%. TB diagnoses were based on the clinical symptoms, sputum microscopy and radiological analyses. TB forms were classified according to TB classification accepted in Russia (instruction no. 109/21.03.2003, Ministry of Health of the Russian Federation). According to this classification, patients were diagnosed with generalized (*n* = 9), fibrous-cavernous (*n* = 1), disseminated (*n* = 15), infiltrative TB (*n* = 21), tuberculosis of the intrathoracic lymph nodes (*n* = 5), exudative tuberculous pleurisy (*n* = 2), cirrhotic TB (*n* = 1), and focal TB (*n* = 4). Disseminated TB was defined as cases with multiple MTB foci preferentially localized in the lungs; fibrous-cavernous TB was identified as the final stage in the development of the earlier forms of lung tuberculosis, characterized by cavities with a strong fibrous capsule internal caseous-purulent layer and common lung damage (instruction no. 109/21.03.2003, Ministry of Health of the Russian Federation). The study was initiated early after hospitalization, with anti-tuberculosis (anti-TB) therapy administered to 14 patients together with antiretroviral therapy, the remaining patients participated in the study before the initiation of TB treatment.

### 2.4. Collection of Sociodemographic Information

Data were collected regarding the level of education (secondary/specialized secondary/University); employment status (work/no work/disability pension); feeling of security (yes/no); marital status (single/married/widow); number of sexual partners during six months preceding the study; number of pregnancies; number of abortions; probable route of HIV infection (self-reported as sexual; through drug use; nosocomial); health damaging habits (smoking, alcohol, use of drugs).

### 2.5. Control Group of WLWH Without Clinical Manifestations of TB

Control group of WLWH without clinical manifestations of TB (WLWH without TB; n = 50) matching in age the study group (Supplementary Table S1) represented a selection from a cohort of 618 WLWH undergoing the dispensary observation at the Moscow Regional Center for Prevention and Control of AIDS and Infectious Diseases and regularly, at least once a year, visiting a gynaecologist as part of the observation. Demographic and clinical characteristics were taken from the electronic medical records. Absence of clinical manifestations of TB was concluded based on the register data of the health-care provider. CD4+ T cell counts were obtained by two-color flow cytometry using phycoerythrin-anti-CD4 as in the study group. Of 50, 21 patients were in stage 3 and 29 in stages 4A to 4C (classification of HIV/AIDS phases adapted by the Ministry of Health of the Russian Federation). WLWH in stages 3 and 4 of HIV-1 infection differed in age (average of 33.5 ± 7.7 years in stage 3, and 37.7 ± 5.9 in stage 4; *p* = 0.016), duration of ART (in years) (1.7 ± 1.6 versus 3.2 ± 2.3; *p* = 0.044) and nadir CD4+ T cell counts (362 ± 207 versus 211 ± 218, *p* = 0.005), respectively. Control group was different from the study group in higher proporion of WLWH on ART, higher percentage of women with CD4 + T cell counts >399, and lower percentage of women with CD4+ T cell counts ≤67 by the time of analysis (*p* < 0.01; Supplementary Table S1). Sociodemographic data of the control group in relation to HR HPV status forms a part of a larger ongoing cohort study of WLWH [35], to be presented in full after its conclusion.

### 2.6. Analysis of Cervical Smears for the Presence of HR HPVs

Smears from the cervical canal, transition zone and exocervix of the patients in the study, and in the control groups, were taken using a disposable brush. The brush together with the material was placed in a container with a stabilizing solution (BD SurePath system, Franklin Lakes, NJ, USA). Isolation of DNA from the collected material was done manually using “Isolation of DNA/RNA on MPs of the SileksMagNA ™ type” kit (Sileks, Moscow, Russia). HPV typing was performed by PCR using the AmpliSens^®^ HR HPV genotype-EPh kit (Interlabservice, Moscow, Russia) with electrophoretic detection of HR HPV types 16, 18, 31, 33, 35, 39, 45, 59, 52, 56, 58, and 66. DNA isolation and PCR analysis were performed according to the protocols of the manufacturers.

### 2.7. Sequencing and Phylogenetic Analysis of HPV 16 DNA

DNA extracted from cervical smears of WLWH co-infected MTB found positive for HPV 16 was amplified with primers specific for genomic regions encoding oncoproteins E5, E6 and E7. The following primers were used: E5-3745 TGC-AAT-TGT-TAC-ACT-TAC-ATA-TG (forward), E5-4318 ATG-TAC-CTG-CCT-GTT-TGC-ATG (reverse), E6-53 GAA-ACC-GGT-TAG-TAT-AAA-AGC-AGA-C (forward), E6-533 AGC-TGG-GTT-TCT-CTA-CGT-GTT-CT (reverse), E7-478 CCA-TAA-TAT-AAG-GGG-TCG-GTG-GA (forward), E7-923 TTT-TTC-CAC-TAC-AGC-CTC-TAC-AT (reverse); primers were described earlier by van Duin et al. [36]. The conditions of PCR reaction were the same for all primer pairs: 40 cycles, enzyme activation at 95 °C for 2 min, denaturation at 95 °C for 30 s, annealing of primers at 58 °C for 30 s, elongation at 72 °C for 2 min and the final elongation at 72 °C for 7 min. Plasmid pHPV 16 containing whole HPV 16 genome (ATCC^®^ 45113D™; ATCC, Manassas, Virginia, USA) served as a positive control. Reaction mixtures were analyzed on a 1.5% agarose gel. DNA fragments of the size corresponding to DNA encoding E5, E6, and E7 were purified from the gel and sequenced on ABI 3500 sequencer using The BigDye^®^ Terminator v3.1 Cycle Sequencing Kit (ThermoFisher Scientific, Waltham, MA, USA).

GenBank was searched for the complete genomes of HPV 16 strains from Europe and USA deposited in the period 2017–2020, and 3320 sequences were identified. The reference sequences from the ICTV classification for alphapapillomavirus 9 were used as a comparison group. All sequences had >98% of identity to the ones obtained in the current study. GeneBank sequences were cropped leaving only E5 and E6–E7 encoding regions, and subpopulations of sequences were selected that differed from each other by at least one encoded amino acid residue (cut-off at at least 0.3% divergence). Phylogenetic trees were constructed for these E5 and E6/E7 encoding sequences and the ones obtained in this study using the maximum likelihood (ML) method realized in the PhyML 3.0 package. The optimal model was chosen using jModelTest2 employing the nucleotide evolution model HKY85, with a heuristic search through tree space NNI, and aLRT SH-like tree quality assessment. The resulting tree was visualized using FigTree v1.4.3.

### 2.8. Cytological Examination of Cervical Smears of WLWH with TB

Another independent collection of smears from endo- and exocervix was performed at a later occasion if patients agreed (10 of 58). The material was taken using a standard cervical brush, applied on the glass in a thin layer and transported to the laboratory, where the smear was stained after the Romanovsky–Giemsa method. The dried cytological preparations were fixed in methanol for three to five minutes, then kept in a ready-made solution of the Romanovsky–Giemsa dye for five to seven minutes, after which they were washed with water, dried, and microscoped. Results were reported by the local pathologist according to The Bethesda System 2014: negative for intraepithelial lesion or malignancy (NILM); atypical squamous cells undetermined significance (ASC-US); low grade squamous intraepithelial lesion (LSIL); atypical squamous cells cannot exclude HSIL (ASC-H); high grade squamous intraepithelial lesion (HSIL); AGC (atypical glandular cells); and squamous cell carcinoma (SCC) (https://bethesda.soc.wisc.edu/index.htm) (30 March 2021). Inflammatory cervical smears without epithelial cell abnormality and reactive cellular changes were also reported. Slides were scanned using Vision Pro Cell Imaging Analyzer (West Medica, Wiener Neudorf, Austria) and the gallery of full slide images viewed by Vision Slide Viewer program (West Medica) was re-evaluated by an independent cytopathologist with confirmation of the initial results. LSIL type was detected in two women. One, infected with HPV 33 and 52, had infiltrating TB and a concomitant diagnosis of candidal esophagitis and CD4+ counts of 400 cells/μL. The other, infected with HPV 16, had tuberculosis of the thoracic lymph nodes and concomitant diagnosis of chronic hepatitis C and CD4+ counts of 55 cells/μL. The remaining eight were negative for intraepithelial lesions or malignancies.

### 2.9. Statistical Analysis

Data analysis was performed using the IBM SPSS Statistics 20.0 software and in part, using nonparametric Mann–Whitney, Kruskal–Wallis, and Spearman rank correlation tests (Statistica 11, Tibco, PaloAlto, CA, USA). Analysis of the level of significance for the difference between two proportions from two independent samples and for the means of two independent populations was done using two-sided difference tests for independent samples (*t*-tests; Statistica 11 platform).

## 3. Results

### 3.1. Clinical Parameters of Women Living with HIV-1 with Tuberculosis

The study enrolled 58 women living with HIV with an established diagnosis of tuberculosis (WLWH with TB). Most were 31–39 years old (58.6%; Table 1). For 96.6% of women, the TB diagnosis was first made in the TB clinics performing the current study (i.e., they did not know they were infected before turning for medical help). Clinical forms of TB were classified according to TB classification accepted in Russia distinguishing more than 10 different forms of TB based on the clinical and morphological criteria, including generalized, infiltrative, fibrous-cavernous, disseminated TB tuberculous pleurisy, cirrhotic TB, and caseous pneumonia, as well as extrapulmonary TB forms. We have scored the severity of clinical manifestations of TB following an experimental approach described by Panteleev AV et al. [37] with the most severe TB forms getting score of 1, and the least severe, score of 7. The most severe clinical manifestations of TB were observed in the patients with generalized (n = 9) and progressive fibrous-cavernous pulmonary TB (*n* = 1) who were assembled into Group 1 (*n* = 10). The rest of patients with other TB forms were assembled into Group 2 (*n* = 48).

**Figure 1:**
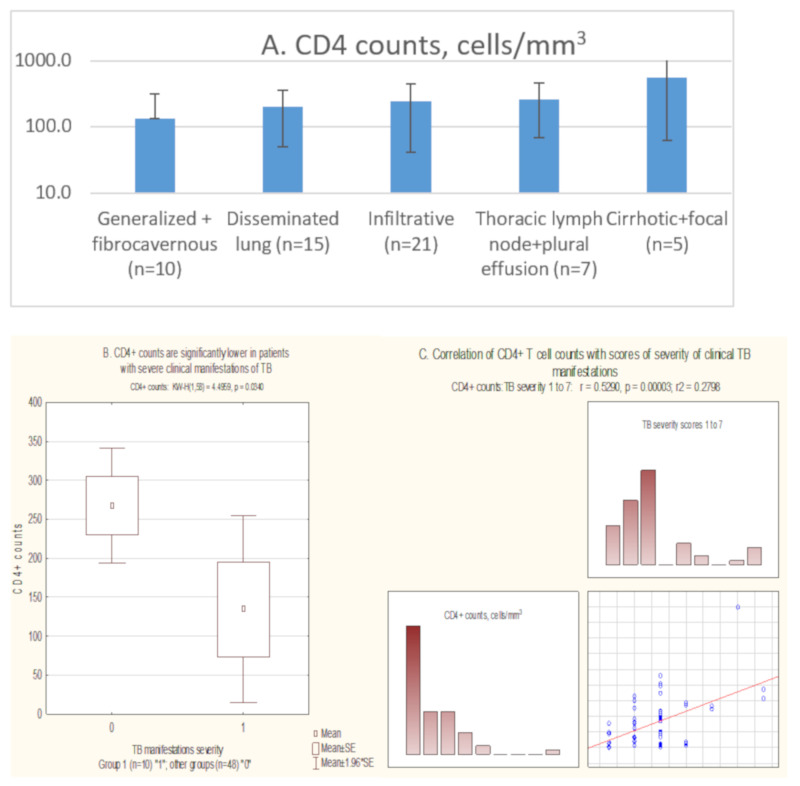

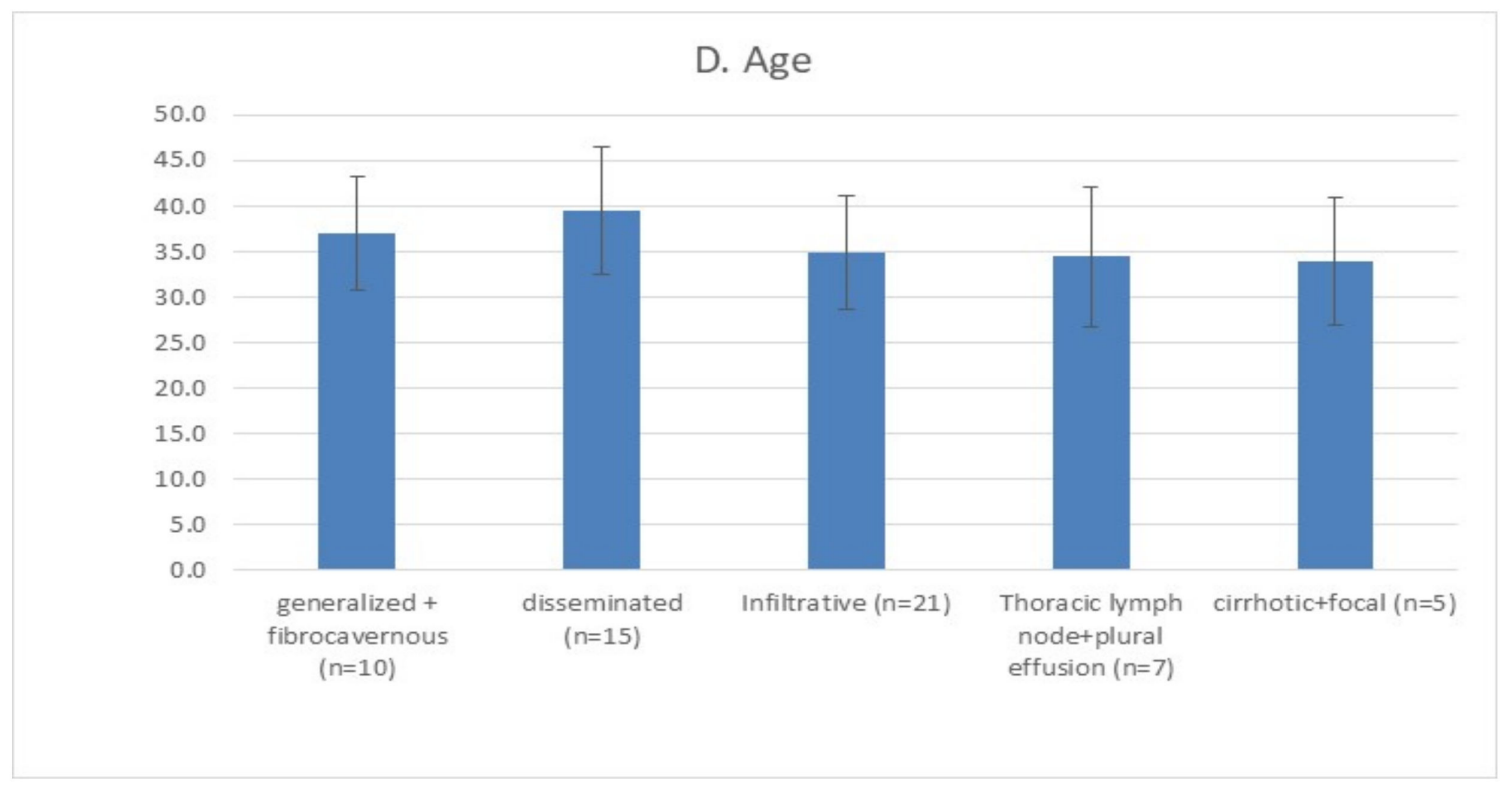
Determinants of the severity of clinical TB manifestations in women living with HIV-1 co-infected with *M. tuberculosis*. Severity of clinical TB manifestations increased with the decrease of CD4+ T cell counts (**A**), with the lowest CD4+ counts observed in patients with severe clinical manifestations of TB (*p* < 0.05) (**B**); CD4+ T cell counts (*y*-axis) and the severity of clinical manifestations of TB, scored as generalized = 1, disseminated = 2, infiltrative = 3, lymph nodes and other = 4, pleural = 5, cirrhotic = 6, and focal = 7 (*x*-axis), were correlated (**C**); The severity of clinical manifestations of TB was not dependent on the on the patient’s age (**D**). Statistical analysis was done using Kruskall–Wallis, Mann–Whitney tests, and Spearman ranking test, and graphically represented using Statistica 11 software; *p* values < 0.05 were considered significant.

Patients with different TB forms differed in their CD4+ T cell counts (Figure 1A; *p* < 0.05). Patients with severe clinical manifestations of TB had significantly lower CD4+ counts compared with other patient groups (Figure 1A,B; *p* < 0.05). CD4+ T cell counts significantly correlated with the severity of clinical TB manifestations (R = 0.529, *p* = 0.00003, Spearman ranking test; Figure 1C). The severity of clinical TB manifestatins did not depend on the age of the patients, duration of HIV infection, or duration of ART for patients receiving treatment (*p* > 0.05; Figure 1D).

On admission to the clinics, 23 of 58 patients were on ART for 2 to 15 years, and the rest (35) were not treated. Of these, 19 (32.7% of the study group) did not know that they were HIV-1 infected and 16 (27.6%) refused treatment. All patients admitted to the clinics were offered to start/continue ART.

The HPV study, including the survey and collection of cervical smears, was performed shortly after hospitalization. By the time of the study, 23 patients continued ART; 8 have started and were on treatment >1 month; 2 started and stopped ART due to drug intolerance; 14 have not yet started or started ART ≤1 month ago, and 5 refused treatment (Table 1). For the purpose of this study, all patients receiving ART ≤1 month (6) were considered as treatment-naïve, as short-term ART therapy was not expected to have a substantial effect on their health. Indeed, ART-naïve women and women on ART for ≤1 month did not differ in CD4+ counts (*p* > 0.05). Thus, by the time of HPV study, 31 patients were considered as ART-treated, and 27 as untreated (Table 1). The median age of WLWH with TB receiving and not receiving ART did not differ (35.8 and 34.8 years, respectively; *p* > 0.05).

By the time of HPV study, TB therapy was administered to 5/10 patients (50%) in Group 1 with severe clinical manifestations of TB. Most of the patients in Group 2 with relatively mild forms of TB such as exudative pleurisy, cirrhotic and focal TB had not yet started either TB-therapy, or ART (39/48; 81.2%). Nine (18.8%), including two with disseminated and five with infiltrative TB, received TB-therapy and ART (Table 1). The average duration of TB therapy in Groups 1 and 2 did not differ (3.8 ± 1.8 versus 3.2 ± 1.3 month, respectively; analysis of the difference between TB therapy duration in Group 1 (5 treated) and Group 2 (9 treated) by Mann–Whitney U Test with continuity correction generated adjusted Z = 0.426615; *p* value = 0.6697; and 2* sided exact *p* value = 0.6993. In patients treated for TB (*n* = 14, Table 1), CD4+ T cell count positively correlated with the duration of ART and of ART+TB treatments indicating gradual immune restoration (R = 0.43, *p* = 0.015; and R = 0.53, *p* = 0.05, respectively; Spearman ranking test).

### 3.2. Sociodemographic Parameters of Patients with HIV/TB Co-Infection

**Figure 2:**
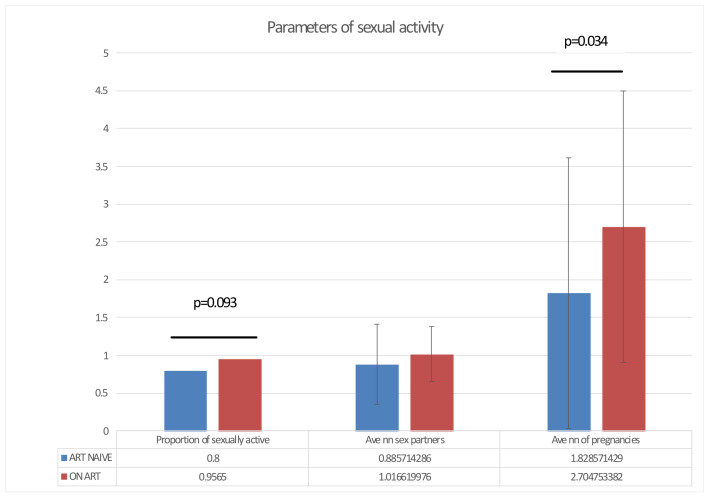
Parameters of sexual activity in WLWH with TB naive to antiretroviral treatment (ART; *n* = 23) and receiving ART by the time of the survey (*n* = 35), including proportion of WLWH self-reporting as sexually active, the average number of reported sexual partners during six months preceding the survey, and the average reported number of life-time pregnancies. Statistical analysis was done by F-test (Statistica 11).

Most of WLWH with TB participating in the study (87.9%; 51/58) was not married; 77.6% reported one sexual partner in 4–6 months preceding sociodemographic survey and HPV testing; 13.8% reported no sexual contacts in the last six month (in majority, 12.1%, in several years) prior to the survey. According to the survey, 69% (40) women had children; 53.4% (31) had 3 or more pregnancies; 34.5% (20) had 1 or 2 abortions (Table 1). Comparison of treatment-naïve women and women on ART showed that women on ART had significantly more pregnancies than untreated women (*p* < 0.05) and tended to be sexually more active than WLWH with TB not receiving ART (*p* < 0.1; F-test) (Figure 2). The opposite was also true: women who had spouses, were more often on ART, and duration of ART in this group tended to be longer than for women who had no sexual partners (*p* < 0.1).

The majority (86.2%; 50) of WLWH with TB had secondary or secondary professional education; 5 (8.6%) of women had completed and 3 (5.2%) had incomplete university education (Table 1). Fewer than one-third of respondents (32.8%; 19) had a job; the rest 39 (67.2%) did not work (Table 1). Of the latter, 10.3% had disability pension, 58% lived on casual earnings and/or were supported by relatives (Table 1). Nevertheless, the majority of the respondents (82.8%) perceived their life/life conditions as secure.

With regards to the means of financial support, 43.5% (10/23) women on ART worked, whereas only 22.9% (8/35) were working among treatment-naïve women (*p* = 0.09). The same was true for the women receiving TB-therapy (TB-therapy+ART): 50% (7/14) receiving ART+TB therapy were working, among untreated women the percentage of working was significantly lower (27%, or 12/44; *p* = 0.02). Altogether, this indicated that women on ART and ART+TB therapy were in a better health condition and/or were feeling better (could work and have sexual relations) than treatment-naïve women.

Risk factors for acquiring HIV-1 were identified in 98.3% (57) patients. An important risk factor was injection drug use reported by 17.2% of study participants (of note, according to the quest drugs were used by 25.9% (15) of the study participants; Table 1). The majority of WLWH with TB (46/58; 79.3%) reported heterosexual mode of HIV-1 transmission (Table 1), however, at least four out of these 46 also practiced injection drug use. Altogether, use of drugs and alcohol and smoking were practiced by 63.8%; almost 27.6% reported more than one of these risk habits (Table 1).

Overall, the sociodemographic portrait of an HIV/TB co-infected woman was as following: age over 30, with secondary education, unemployed, having a sexual partner without marriage, with two or three pregnancies and at least one abortion, and a smoker. Absence of the knowledge of HIV and TB status, refusal of ART for those knowing their HIV+ status, along with several health-damaging habits, indicated indifference of the majority of study participants to their health.

### 3.3. Prevalence of HR HPVs Among Women Living with HIV-1 with or without TB

**Figure 3:**
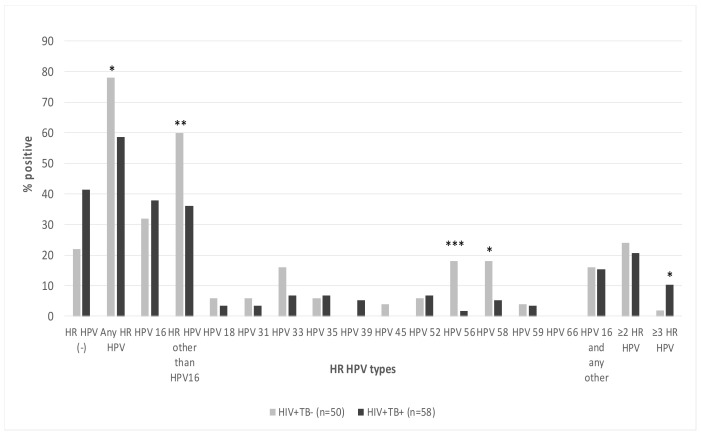
Prevalence of HR HPVs in the cervical smears of women living with HIV-1. TB -negative WLWH (*n* = 50) and WLWH with TB (*n* = 58). * *p* = 0.03, ** *p* = 0.014, *** *p* = 0.0035, two-sided difference test (Statistica Axa 11).

Cervical smears of 41.4% of HPV study participants (24/58) were negative for all 12 HR HPVs. HPV 16 was detected in 38% (22/58), i.e., in the majority of positive samples (64.7%; 22/34). Other HR HPVs were detected in 34.5% (20/58) of WLWH. The prevalence of HR HPV genotypes other than HPV 16 (HPV 18, 31, 33, 35, 39, 52, 56, 58, and 59) among WLWH with TB was low (1.7 to 6.9%; Figure 3). HR HPVs of one type were detected in 37.9% of women; 20.7% (12/58) were infected with two or more HR HPV types (Figure 3). One patient was infected with three types of HR HPVs (HPV 16, 18, and 52). Thus, in women with HIV/TB co-infection, the dominant HR HPV type was HPV 16.

Interestingly, control group of TB-negative WLWH demonstrated higher HR HPV prevalence (Figure 3). Besides the fact that TB-negative WLWH had higher prevalence of HR HPVs, more women were co-infected with HR HPVs other than HPV 16, especially HPV 56 and HPV 58 (18% for each compared to 1.7% for HPV 56 and 5.2% for HPV 58 in case of WLWH with TB; *p* < 0.05) (Figure 3). One patient was infected with four HR HPV types (HPV 35, 45, 56, 59; the only one among totally 108 WLWH found to be infected with HPV 45). Five WLWH were co-infected with three HR HPV types. While the number infected with two HR HPVs did not differ, the number infected with >2 HR HPV types among TB-negative WLWH was significantly lower than among WLWH with TB (1/59 vs. 6/50; *p* = 0.03; Figure 3). Thus, in both WLWH with and without TB, the dominant HR HPV type was HPV 16. The overall prevalence of HR HPV infection was higher in TB-negative WLWH (*p* = 0.03), but the diversity of HR HPV types was less pronounced than in WLWH with TB.

Out of 58 women in the study group, only 10 agreed to undergo repeated sampling with cytological examination of the cells in the cervical canal for morphological changes. LSIL was detected in two women, one infected with HPV 16 and the other, with HPV 33 and 52. Other patients (8/10; 80%) were negative for the intraepithelial lesions or malignancies.

### 3.4. Phylogenetic Analysis of HPV 16 and Characteristics of E5, E6, and E7 Oncoproteins of HPV 16 in WLWH with Tuberculosis

The quality of the samples and HPV viral load allowed us to obtain sequences of E5, E6 and E7 encoding DNA from 11 out of 22 HPV16-positive samples of WLWH with TB. The resulting sequences were entered into the international NCBI GenBank database and assigned numbers MT353690-MT353699, MT353701-MT353712. Their analysis revealed a low level of divergence. The phylogenetic trees based on E5 and on E6/E7 demonstrated that they did not have a common ancestry and were similar to E5, E6, and E7 from HPV 16 isolated in other European countries and the USA reflecting diversity of the circulating HPV 16 strains (Supplementary Figure S1A,B).

E5, E6, and E7 oncoproteins sequences were found to be highly conserved, specifically at the amino acid level. The most conserved was the E7 oncoprotein, in which only one substitution on amino acid level was observed, namely N29S in the GeneBank sequence MT353705. The rest of the E7 sequences were identical to the reference HPV 16 variant NC_001526. More polymorphisms were found in the E6 oncoprotein. E6 oncoprotein contained several single amino acid substitutions unique for each of the samples. Besides, 5 out of 11 samples contained substitutions R17G and L90V (GeneBank accession nn MT353702, MT353703, MT353707, MT353709, MT353710), and MT353708, single substitution R17G. For the E5 oncoprotein, a single amino acid substitution I65V was recorded in two samples; other sequences were identical to the NC_001526 reference. Thus, main HPV 16 oncoproteins identified in WLWH with TB did not differ from that circulating in other population groups around the world.

### 3.5. Demographic and Clinical Characteristics Associated with HR HPV Infection in WLWH with Tuberculosis

Firstly, we analyzed prevalence of HR HPVs WLWH with TB in relation to the objective characteristics, such as age, immune status (CD4+ T cell counts), duration of life with HIV, severity of TB infection, and ART and TB therapy.

#### 3.5.1. Age

The prevalence of HPV 16 was higher in the age group <29 years (*n* = 8) than in other age groups (Table 2). Infection with other HR HPVs did not differ between the age groups (*p* > 0.1; Table 2). Besides, in women aged <29, there was a tendency to more frequent infection with HPV 16 together with other HR HPV types than in the other age groups (*p* = 0.07). No age dependence of the prevalence of HR HPVs was observed in the control group (Supplementary Figure S2A,B).

#### 3.5.2. Immune Status

Infection with HR HPVs in the study and in the control groups was not correlated with CD4+ counts (Table 2; Supplementary Figure S3A). However, in the study group, there was a trend towards inverse correlation between CD4+ counts and the number of HR HPV types detected in the cervical smears (*p* = 0.0651). Also, in the study group, HPV 16 tended to be more frequent among patients with CD4+ counts <200 cells/mm^3^ than in those with CD4+ >350 cells/mm^3^ (*p* = 0.059), while the prevalence of other HR HPVs was not related to the immune status (Table 2). In the control group, there was no difference between prevalence of any of HR HPV types alone or in combination according to stages of HIV-1 infection, or CD4+ T cell counts by the time of analysis (WLWH were grouped as having >399, 200–399, <200, or <67 CD4+ T cells per mm^3^) (Supplementary Table S2; Supplementary Figure S2C).

#### 3.5.3. Duration of Life with HIV-1

WLWH with TB positive and negative for any HR HPV, HPV 16, or any other HR HPV of the 12 tested, or ≥2 HR HPV types, did not differ in the duration of life with HIV-1 (in years or month; *p* > 0.2). The number of infecting HR HPV types detected in cervical smears of WLWH with TB was not correlated to the years of life with HIV-1 (R = 0.18, *p* > 0.2; Spearman ranking test). On the contrary, WLWH without TB showed dependence of the number of detected HR HPV types on the duration of life with HIV-1 (Supplementary Figure S2A). Significance of the latter correlation could be explained by the higher overall positivity for HR HPVs in WLWH without compared to WLWH with TB, providing sufficient data to run a correlation test.

#### 3.5.4. TB Infection Forms and TB Severity

**Figure 4:**
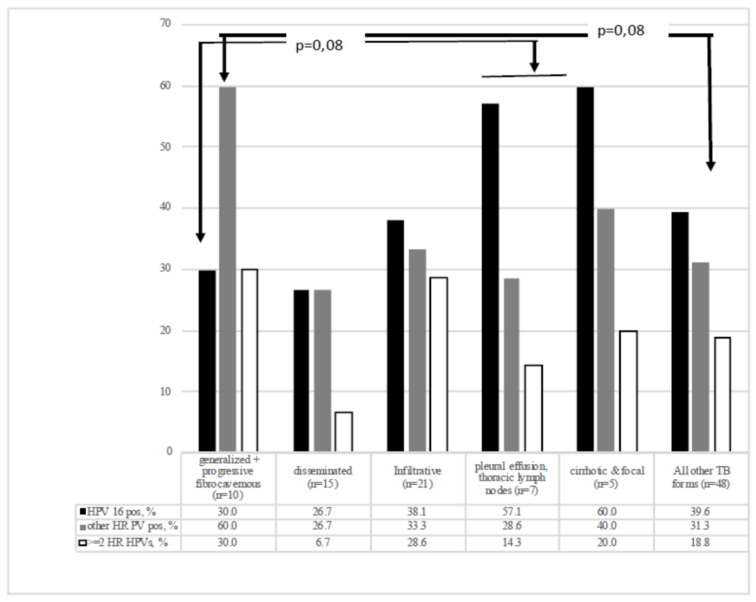
Prevalence of infection with HR HPVs among WLWH with different clinical forms of tuberculosis (*n* = 58).

WLWH with severe clinical forms of TB (Group 1) exhibited relatively low prevalence of HPV 16 (30%) and high prevalence of other HR HPVs (60%), whereas patients with other TB forms specifically cirrhotic, focal and extrapulmonary TB, on contrary, tended to exhibit higher prevalence of HPV 16 (60%) and lower prevalence of other HR HPVs (30% for patients in Group 2, although the difference in HR HPV prevalence between groups with different TB forms did not reach the level of significance; *p* = 0.08; Figure 4). The TB form/severity of clinical manifestations of TB infection had no significant effect on the proportion of patients infected with ≥two types of HR HPVs (Figure 4).

#### 3.5.5. Antiretroviral Therapy

Prevalence of infection with HR HPVs in general and with HPV 16 in particular did not depend on whether the patients were on ART or not. There was no difference in the prevalence of HPV 16 infection in WLWH with TB receiving NRTI + NNRTI, or NRTI + PI, and those who did not receive treatment (Supplementary Figure S3A,B; Supplementary Table S3). On contrary, HR HPVs other than HPV 16 were significantly more common among WLWH with TB receiving ART (*p* = 0.02; Table 2; Supplementary Figure S3C). The rate of detection of HR HPVs other than HPV 16, and the number of HR HPV types, specifically HR HPVs other than HPV 16, were significantly lower in treatment-naive patients compared to patients on ART independently of the treatment regimen (2 NRTIs + 1 (2) NNRTIs, or 2 NRTIs + 1 (2) PIs) (see Supplementary Figure S3D,E for illustrations and statistics). WLWH in the control group exhibited no dependence of HR HPV prevalence on ART or its duration (Supplementary Table S4). Absence of difference might have been linked to too few participants in the control group who were naïve to ART (90% were on ART for ≥3 month).

#### 3.5.6. TB Therapy

Anti-TB treatment had no effect on the prevalence of HPV 16 (Table 2). Prevalence of infection with HR HPVs other than HPV 16, and of simultaneous infection by two or more HR HPV types was significantly higher among WLWH with TB receiving anti-TB therapy compared to naïve to TB treatment (*p* < 0.05; Table 2), i.e., followed the trend observed for ART. ‘Non-16′ HR HPVs may have been more often detected in patients on TB therapy than in those who were not due to the fact that patients receiving TB therapy were also on ART. The significance of difference between prevalence of HR HPVs in TB-treated versus TB-untreated patients was, however, higher than significance of difference between those receiving or not receiving ART (Table 2), suggesting that TB-therapy was an independent factor associated with high prevalence of ‘non-16′ HR HPV types.

**Figure 5:**
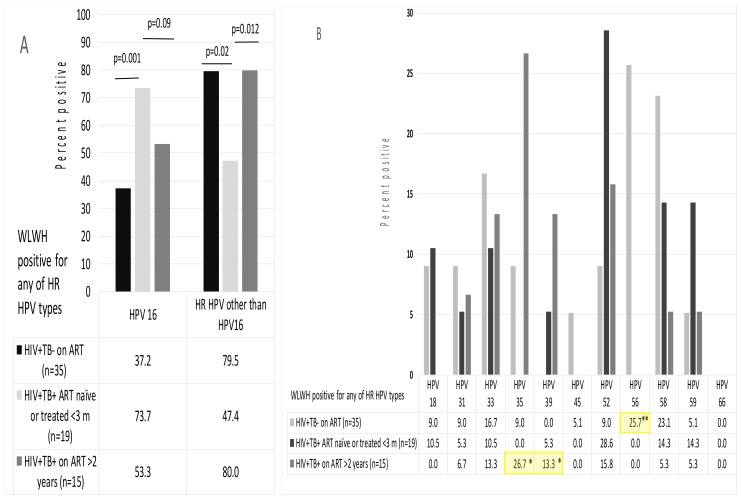
Representation of HR HPV genotypes in HR HPV positive cervical smears. WLWH positive for any of HR HPVs represented by WLWH without TB on ART (n = 35), WLWH with TB naïve to ART or ART treated <3 month (treatment started in the hospital; n = 19) and WLWH with TB receiving ART for >3 month (on treatment before hospitalization; n = 15) were compared in prevalence (%) of HPV 16 and of any HR HPV other than HPV 16 (**A**), and of individual HR HPV genotypes (**B**). * *p* < 0.05, WLWH with TB compared to WLWH without TB for those ART-treated for >3 month; ** *p* < 0.05 WLWH without TB on ART compared to both subgroups of WLWH with TB; two- sided difference test (Statistica 11).

We have further assessed the distribution of HR HPVs in the positive samples. HPV 16 was detected in 53% of HR HPV positive samples of WLWH with TB and 37% samples of WLWH without TB, the difference not reaching the level of significance (*p* = 0.09); ART-naïve WLWH with TB were characterized by significantly higher prevalence of HPV 16 (Figure 5A). At the same time, WLWH without TB (*n* = 45) and WLWH with TB receiving ART >3 month (those on ART before hospitalization *n* = 15) demonstrated similar high prevalence of HR HPVs other than HPV 16; in treatment naïve WLWH with TB it was almost two times lower (79.5–80% versus 47.4%, *p* < 0.05; Figure 5A). Distribution of 11 other HR HPV genotypes was more even. WLWH with TB on ART >3 month demonstrated increased prevalence of infections with HPV 35 and 39, and WLWH without TB receiving ART, increased prevalence of HPV 56; distribution of other HR HPV genotypes among samples positive for HR HPV DNA did not differ (Figure 5B).

Thus, HR HPV prevalence and distribution among WLWH with and without TB have many common features, such as high overall positivity for HR HPVs (>60%), dominance of HPV 16, and frequent (in up-to 20%) positivity for two or more HR HPV types. However, we observed a marked difference in the prevalence of HR HPVs other than HPV 16. In WLWH with TB their prevalence was two-times lower than in TB-negative WLWH. Furthermore, it was positively associated with ART: in ART treated WLWH, prevalence of HR HPV types other than HPV 16 was nearly two-fold higher than in ART-naïve patients, while prevalence of HPV 16 was lower (*p* values < 0.05). This scenario was valid for both WLWH with and without TB (although CD4+ T cell counts in WLWH without TB were significantly higher; Supplementary Table S1). These data indicated that antiretroviral treatment in both patients with and without clinical manifestations of TB leads to an increased prevalence of HR HPVs other than HPV 16. This questions (direct) association of high prevalence HR HPVs in WLWH with immune suppression, indicating presence of other and/or additional underlying factors. We looked into the socio-behavioral characteristics of WLWH with TB to identify if they could help to explain the observed HR HPV prevalence.

### 3.6. Socio-behavioral Characteristics of WLWH with TB Influencing HR HPV Infection

#### 3.6.1. Education

The prevalence of infection with HR HPVs, both HPV 16 and ‘non-16′ types, did not depend on the level of education graded as ‘secondary’ or ‘higher than secondary’ (secondary special, higher and incomplete higher) as well as in more narrow groups (secondary, secondary special, higher/incomplete higher; Table 2).

#### 3.6.2. Occupation

WLWH with TB were divided into two groups:

working (employed or working informally) and not working (receiving disability pension and/or supported by the relatives). Working women were characterized by higher prevalence of infection with ‘non-16′ types of HR HPVs (*p* = 0.039) and multiple HR HPV types (*p* = 0.02; Table 2).

#### 3.6.3. Sexual Activity

We analyzed whether the frequency of HR HPV infection was influenced by the sexual activity. The prevalence of infection with HR HPVs, HPV 16, and other HR HPV types, did not depend on presence or absence of the sexual partner (partners) during six months preceding the survey and testing (self-reported by the study participants; Table 2). At the same time, positivity for HR HPVs depended on the number of pregnancies: those who had ≥2 pregnancies were predisposed to be infected with HR HPVs than those with had <2 pregnancies (*p* = 0.048). This relationship was not observed for HPV 16 or ‘non-16′ HR HPVs taken separately (Table 2). The incidence of infection with HR HPVs was not influenced by the number of abortions.

#### 3.6.4. Risk Factors for HIV Infection

Women who indicated different risk factors for HIV infection (sexual contacts or drug use) did not differ in the prevalence of infection with HR HPVs, or HPV 16, or ‘non-16′ HPV genotypes (data not shown).

#### 3.6.5. Risk Behavior

Smoking versus not smoking, and smoking >5 cigarettes per day, use of alcohol or drugs, separately or combined, had no effect on the prevalence of HR HPVs or the number of infecting HR HPV types (Table 2, and data not shown).

## 4. Discussion

Numerous studies have delineated the effect of HIV-1 infection on infection with HPVs/HR HPVs, development of HR HPV-associated lesions and eventually HPV-associated cancer. On the overall, HIV-1 infection modifies HPV pathogenesis and promotes cancer through multiple mechanisms related to complex HIV-induced immune suppression, dysfunction, and persistent inflammation [15,38,39,40,41], as well as direct oncogenic effects of HIV proteins, also in the cervix [42].

Like HIV, TB induces immunosuppression and promotes tumorigenesis. *Mycobacterium tuberculosis* (MTB) antigens recruit mesenchymal stem cells which repress Th1 immune response and promote lung cancer metastasis [29,43]. In addition, TB induces a hematopoietic shift associated with expansion of myeloid-derived suppressor cells associated with chronic inflammation, most notably in cancer [44]. The potentiation of HR HPV infection by TB and progression of HPV-associated lesions in HIV/TB-coinfected individuals was assumed, but was never addressed in detail during 40 years that passed since the first reports on mucocutaneous HIV/AIDS-related manifestations. We found only one study describing high prevalence of TB infection among WLWH with HPV-associated cervical cancer in Botswana, a low-income country endemic for HIV and TB infections [31].

Here, we performed a study of HR HPV infection in a cohort of women living with HIV-1 with clinical manifestations of tuberculosis (WLWH with TB) residing in Moscow, Russia. Russia is highly endemic for both HIV-1 and TB. Russia is classified today as a middle-income country according to WHO criteria of the health care spending (5% of GDP) [26,45]. The TB-related epidemiological situation in Russia is improving, and currently is reported at 41.2 cases per 100,000 population [46]. Progress is, however, hampered by the expanding HIV epidemic causing an increase in the number and proportion of patients with advanced stages of HIV infection who start ART late. The proportion of such patients has grown from 20.9% in 2017 to 23.1% in 2018 [46]. Consistent with this, the majority of our study participants were admitted to the clinics at late stage of HIV infection classified as 3C/AIDS (stages 4B (53.4%) and 4C (43.1%) according to the Russian classification), with many patients not knowing that they were HIV-positive, and 96% not knowing that they had TB. This scenario fuels both HIV and TB epidemics, and inevitably also the persistence and spread of other chronic viral infections associated with immune suppression, many of which—similar to infection with HR HPVs—may culminate in cancer.

HIV-1 co-infection promotes rapid progression of TB after infection or reinfection as well as reactivation of latent TB [47]. While in HIV-negative individuals, pulmonary TB forms constitute up to 88% and extrapulmonary, 12% of disease manifestations, in people living with HIV (PLWH), the proportion with pulmonary TB decreases two-fold (42%) [48]. At the same time, the prevalence of extrapulmonary disease such as pulmonary granulomas, lymph node tuberculosis (LNTB), pleural effusion, meningitis, urogenital, alongside with disseminated and miliary TB grossly increases [49,50,51,52,53] and can constitute up to 75% of clinical TB manifestations [54]. In the cohort of 58 HIV/TB-coinfected women studied here, only 13.8% had extrapulmonary TB forms resembling disease course in HIV-negative individuals.

Lymph node TB is the most common extrapulmonary TB form in the immunosuppressed individuals including PLWH. It represents 16–30% of extrapulmonary tuberculosis cases [55,56,57], and >40% TB cases in PLWH [58]. In our study, the number of cases of lymph node TB was significantly lower (8,6% of the cases; *p* < 0.05). The second most common site of extrapulmonary TB, occurring in primary as well as reactivation disease, is pleura [59]. In non-HIV endemic areas where reactivation is the predominant mechanism of TB disease, TB pleuritis is reported to occur in only 4%, and in the regions endemic for HIV, in up-to-30% of patients [59,60]. We found pleural TB in Moscow HIV/TB patients to be uncommon (3% of the study group; *p* < 0.05) as in the areas non-endemic for HIV-1. Another common site of extrapulmonary TB is the genitourinary (GU) tract, a primary target of hematogenous TB infections; GU TB comprises 2–14 % of TB cases in the developed and up to 20% cases in the developing countries [61,62]. GU TB commonly affects kidneys as well as the genital tract [63]. HIV/AIDS increases the risk to develop GU TB [58]. In our study, we found GU TB in only one patient. On the overall, prevalence of extrapulmonary TB manifestations (13.8% of TB cases) was significantly lower than recently reported for China, another upper-middle-income country highly endemic for TB (33.4%; *p* = 0.02) [64].

We identified 15 cases of disseminated TB (25.9%) and 9 cases of generalized TB (15.5%) classified as cases with multiple caseous MTB foci preferentially in the lungs or in the lungs and other organs, respectively [65]. HIV-associated disseminated and generalized TB cases are under-recognized and poorly characterized. In HIV-1 endemic settings, disseminated TB with MTB detectable in the bloodstream may constitute up-to-70% of all cases of TB infection [49,66]. In our study cohort, the prevalence of disseminated lung and generalized TB forms was lower than figures reported for HIV/AIDS patients in areas endemic for HIV-1 with a low percent of income spent for health care (*p* < 0.05). Overall, the clinical features of TB in WLWH of the Moscow cohort, with its significant proportion of pulmonary TB forms, followed the clinical features of TB (mono)infection in the middle/high-middle-income countries [45,67]. Important role in this could have been played by BCG vaccination included into vaccination program in Russia. BCG has a protective effect against all forms of TB independent of HIV-1 status, and was shown to confer protection against extrapulmonary TB forms among HIV-negative individuals [68]. Our data indicate that certain level of protection may be provided also for people living with HIV-1. Not less important could be a better overall quality of life of TB patients in the middle-income country, compared to similar cohorts in the low- and low-middle income countries, specifically with respect to nutrition [69]. This argument is supported by the analysis of questionnaire revealing that majority of WLWH in the study group evaluated their life situation as secure.

Next, we performed a systematic analysis of the effect(s) of the clinical manifestations of TB infection in WLWH on the infection with high risk HPV types. MTB-induced immunosuppression/immunomodulation is thought to be limited to the infected organs [43]. In view of this, initiating the study, we hypothesized that the effect of TB on the infection with HR HPVs will depend on the clinical forms and severity of infection, in relation to localization of MTB infection site(s), pulmonary versus extrapulmonary, specifically localization to GU tract. However, due to the prevalence of the pulmonary TB and remote localization of the sites of extrapulmonary MTB infection from the GU tract (the absence of urogenital tract MTB cases), clinical TB manifestations/TB forms had no measurable effect on HR HPV prevalence. An exception was a subgroup of WLWH with generalized TB, different from other TB patients in having significantly lower CD4+ T cell counts, crucial determinants of the severity of clinical TB manifestations (Figure 1). These women exhibited relatively low prevalence of HPV 16 and high prevalence of other HR HPVs (on contrary to patients with higher CD4+ counts characterized by relatively high prevalence of HPV 16 and low prevalence of other HR HPVs).

Low CD4+ T cell counts are thought to predispose to HPV infection and reactivation of HPV infection due to incomplete viral clearance, whereas sustained HIV-1 suppression and higher CD4+ T cell counts are expected to reduce the risk of persistent HR HPV infection [15,70,71]. Here, we could not relate the overall HR HPV prevalence to CD4+ counts except for a tendency of increased prevalence of HPV 16 among WLWH with TB with CD4+ T cell counts <200 cells/mm^3^.

The effect of ART was puzzling: it did not influence the prevalence (infection/persistence) of HPV 16, but increased the cumulative prevalence of other HR HPV types in ART-treated compared to untreated WLWH regardless of their TB status (with certain differences with respect to individual HR HPV genotypes; Figure 4 and Figure 5). Published data on the effect of ART on HR HPV prevalence is controversial. Some studies demonstrated that ART significantly reduces HR HPV prevalence, while others have shown that ART has no effect [15,71,72,73,74,75]. Undoubtedly, on the long term, ART increases life expectancy and with this, potentially increases the duration of HPV infection, which leads to the accumulation of genetic changes at the cellular level, increasing the likelihood to develop cervical cancer [76,77]. At the same time, ART gives benefits in reducing the risk to develop high-grade squamous intraepithelial lesions progressing to cancer [47]. However, one has to keep in mind that ART restores only some of the phenotypic and functional abnormalities associated with HIV infection. Persistent aberrant activation of monocytes, NK, and innate lymphoid cells (ILCs) remains, contributing to the incomplete recovery of T-cell effector functions [78]. Importantly, a recent study by Papasavvas E. et al. revealed that increased levels of immune activation linked to T and myeloid cell exhaustion and immune dysfunction are associated with HR HPV infection, also on the background of successful ART [79]. At the same time, Kelly et al. has shown that an early on-start of ART and better adherence decrease the risk of HR HPV infection and progression to cervical cancer [70]. This indicates the importance of ART not as such, but in the context of the timing of introduction and treatment duration, the prolonged uncontrolled HIV-1 replication irreversibly damaging the immune system and causing accelerated acquisition of co-infections, including HR HPVs. In this study, it is supported by direct correlation of the number of co-infecting HR HPVs types with the duration of life with HIV-1 for TB-negative WLWH (not with their age!) and with high prevalence of HR HPVs in patients with the most severe clinical TB manifestations characterized by gross longitudinal dysfunction of the immune system.

This concept can explain the overall HR HPV prevalence, but it does not explain a difference in the prevalence of HPV 16 and cumulative prevalence of other HR HPV types. Few studies described competition between HPV 16 and other HR HPVs [80,81,82]. This scenario implies that HPV 16 infection/re-infection is suppressed (or excluded) by infection with HR HPVs of other genotypes. Indeed, certain HR HPV types were prevalent in ART treated WLWH without TB (HPV 56) and others, in ART treated WLWH with TB (HPV 35, 39), indicating systemic loss of the viral competition by HPV 16 (failure) in favor of other HR HPVs, i.e., of ART giving a privilege to infections with HR HPVs other than HPV 16.

We looked for explanations of this ‘privilege’ — prevalent infections with HR HPVs other than HPV 16 among women receiving ART and ART/TB-treatments — in their demographic features and socio-behavioral profiles. Similar to previously published studies, we observed that HR HPV infection in WLWH with TB was age-dependent. In the developed countries, the highest percentage of HR HPV infected persons is detected in the age group ≤25 years [83]. Similarly, in our study, the highest prevalence of HR HPVs (87.5%) and of HPV 16 in particular (75%) was detected in the age group <29 years (Table 2). This is in lines with the earlier observations by our group [35] and by others [84] that the prevalence of HR HPVs (and of cervical abnormalities) is the highest (up to 50%) among young women, but significantly decreases with increasing age (reaching 20% among women aged over 50 [35]). These findings could be explained by gradual increase with age of the level of anti-HPV antibodies which may hinder HPV re-infection and new infections [85]. The fact that no reduction in the prevalence with age was observed for ‘non-16′ HR HPVs and for infections with multiple HR HPV genotypes (Table 2) might result from the encounters with new (previously not encountered) HR HPV variants.

Certain role in HR HPV competition could have been played by varying levels of sex hormones. Estrogen increases the expression of HPV 16 genes (although mechanisms remain unclear) and provides a growth advantage to HPV 16 positive cervical cell lines protecting them from apoptosis [86]. With this, HPV 16, highly common and relying on the high level of expression of sex hormones, represents an infection of the sexual debut. Aging results in fluctuating and eventually decreasing levels of ovarian estrogens creating a less advantageous environment for HPV 16 infection [87]. In this scenario, as in the first one, the older age could be a factor pre-disposing to the infection with HR HPVs other than HPV 16. This, however, cannot explain our data on the prevalence of these HR HPVs in WLWH on ART independent of their age, and not in WLWH with generalized TB that were not older than patients with milder clinical TB manifestations (there was no age difference in patients with different severity of clinical TB manifestations; *p* >0.5).

To find an explanation, we compared the sociodemographic portrait of our cohort of WLWH with TB with a portrait of WLWH made in an earlier study of 7000 patients, including 49% women, followed up in 27 Russian AIDS centers during 2014 [88] and with the latest data from the AIDS centers of 85 territorial subjects of the Russian Federation [89]. The major social and demographic indicators of our cohort matched those described by Pokrovskaya et al. [88] and Ladnya et al. [89], strengthening the value of our observations. In agreement with the data of these studies [88,89], the prevalent mode of HIV transmission in our HPV cohort was heterosexual (79.3% of respondents here and 77.4% by Pokrovskaya et al. [88]). However, the route of HIV-1 infection (indicating un-protected sex) had no effect on the prevalence of either HPV 16, or of other HR HPV types. Furthermore, the prevalence of HR HPV infections and number of infecting HR HPV types was not associated with the number of sexual partners self-reported by the participants, and not with the use of drugs and/or alcohol known to increase sexual activity and risky sexual behavior, altogether minimizing the role of risky sexual behavior, in HR HPV infection of WLWH with TB in the period preceding this study.

Smoking is a known risk factors for HPV [30,90] and for TB infection [91,92] as nicotine metabolites suppress local immunity. Out of the 58 participants in our study, 55.2% smoked; this figure exceeds the national average two-fold—according to the Russian Public Opinion Research Center data, in 2019, regular consumption of nicotine-containing products is practiced by 33% of the adult population of Russia, with women accounting for 25% of the smokers [93]. However, neither smoking as such, nor the number of smoked cigarettes per day (exceeding five in all smokers) had any effect on the prevalence of HR HPVs or the number of HR HPV types infecting the cervix. The overall effect of the substance use (smoking, alcohol, drugs) on the prevalence of HR HPVs in WLWH with TB was negligible.

In this unique cohort, we found only two sociodemographic parameters that were associated with the prevalence of infection with HR HPV. One was the number of pregnancies (Table 2). HR HPVs were detected more often in women with ≥2 pregnancies compared to women with one pregnancy and to women who was never pregnant (*p* < 0.05). Interestingly, women who have had three or more pregnancies were earlier reported to have an increased risk of developing cervical cancer [94,95]. Also, the rate of development of HSIL was found to correlate to the number of full-term pregnancies, although no direct associations were found between the development of HSIL/cervical cancer and serological markers of HPV infection [96]. The lead risk factor to develop HSIL and cervical cancer are the hormonal changes during pregnancy. Recent studies in HPV transgenic mouse models provide evidence that estrogen and its nuclear receptor promote cervical cancer in combination with HPV (majorly HPV 16) oncogenes [97]. The steroid hormones modulate HPV gene transcription [98,99], and can therefore enhance the expression of viral oncogenes in persistent HR HPV infection in the settings of pregnancy, eventually leading to malignant transformation of the expressing cells, with associations between estrogen and HPV-positive cancers supported by clinical evidence [100]. Our finding of the association of the number of full-term pregnancies with HR HPV prevalence support the role of sex hormones in HPV persistence, translated into higher HR HPV detection rates, however, irrespective of HR HPV types (Table 2), thus, was not explaining prevalence of certain HR HPV types (as WLWH positive for HPV 16 or for other HR HPVs did not differ in the number of pregnancies; Table 2).

The number of sexual partners is a well-known risk factor for acquiring HPV infection [101,102]. Prevalence of HPV infection with HR HPVs was shown to double in women with two sexual partners compared to women who had one [102]. Furthermore, up to 14% of HPV DNA in the cervix may appear not due to the actual HPV infection, but due to the temporary deposition (‘colonization’) by the infected semen, detectable for up to 15 days post-unprotected sex [103]. In our group, we could not show any depedence of HR HPV prevalence on the self-reported number of sexual partners (Table 2). However, one has to keep in mind that in confidential non-anonymous quests women report a reduced number of sexual partners compared to the anonymous testing [104]. The role of sexual HPV transmission in WLWH with TB is indicated by the number of pregnancies as an objective indicator of sexual activity. In support of this option, WLWH with severe clinical manifestations of TB (Group 1) had statistically more sexual partners during last six months preceding the test than WLWH with milder TB manifestations (*p* < 0.01). We also observed a tendency to a higher prevalence of ‘non-16′ HR HPVs among WLWH with TB who had sex with partners during six months preceding HPV testing.

Second factor associated with HR-HPV prevalence identified in our cohort was occupation. Only 32.8% of our study group worked, the figure being two times lower than that for WLWH study by Pokrovskaya (64%; [88]). Interestingly, women who worked were more often positive for ‘non-16′ HR HPVs (*p* = 0.039) and were more often infected by ≥2 HR HPV types (*p* = 0.02). Both features appeared to be connected to ART or combined ART+TB-treatment: women on ART had significantly more pregnancies than untreated women, and worked more often than treatment-naïve individuals (a tendency for ART-, and significant difference for ART+TB-treatment; Table 2). These observations indicated that on the overall, ART/ART+TB treated women had better physical health than treatment-naïve women, which allowed them to work and to sustain sexual relations. This may also relate to TB-negative WLWH on ART, who have similar high prevalence of HR HPVs other than HPV 16 (Figure 4).

Having this in mind, we returned to our findings of ART/TB-treatment increasing the prevalence of HR HPVs other than HPV 16, and assessed if ART/TB-treatment (in ward) could have modified the lifestyle of the study participants. Indeed, by analysis of patient histories, we found that due to the success of ART and TB-treatments, and improved health of the patients, they were released home on the weekends. In conversations with the doctors, patients admitted that they felt fit to return to their former lifestyle including their sex life. Most of the study participants were under 40, single and led an active life before hospitalization, and could have met their sexual partners during this leave. Several patients met their future husbands while staying at the hospital. This may have resulted in de novo HPV infections and re-infections. This scenario is supported by the latest evaluations of the positive effects of TB treatment on the physical, emotional and psychological health of TB patients [105]. Thus, sociodemographic features of WLWH with TB, suggested an explanation for the increased prevalence of HR HPVs in those receiving ART and ART/TB-treatments in their improved health allowing return to everyday life with capacity to work and upkeep sexual relations. This scenario could be relevant for all WLWH on ART, also those without clinical TB manifestations.

Remaining to address (and explain) was exclusion from this scenario of HPV 16, as its representation among HR HPV positive WLWH on ART was, on contrary, reduced. We hypothesize that women >29 years old may have natural immunity against HPV 16, the infectious agent of sexual debut, restricting re-infections [85], especially in the context of ART-induced immune restoration. Other HR HPVs could be de novo types which WLWH have not earlier encountered, resulting in preferential ‘non-16′ HR HPV infections. An interesting finding in the context is a gender disparity between HR HPV types among sexually active adolescents and young adults in Brazil, with HPV 59 prevalent in men, and HPV 16, in women (despite introduction of HPV vaccination of girls in 2014) [106]. Another disparity between HR HPV types was uncovered in relation to the geographic origins of the study participants, with HPV 52 prevailing among Chinese women living in urban, and ‘old-fashioned’ HPV 16, among women living in the rural areas [107]. The latest data by Wu W et al. demonstrated an increase in the prevalence in China of HR HPV of types 52 and 58, prevailing over HPV 16 [108]. Of note, China has not yet adapted HPV vaccination program, meaning that the above data is not a result of the implementation of HPV vaccination, but rather a reflection of a longitudinal shift in HR HPV prevalence caused by accumulating immunity of population to previously dominant HR HPV types, as HPV 16. This shift would explain high prevalence in the cohort of WLWH on ART of ‘non-16′ HR HPVs, specifically in sexually active individuals. We are currently verifying this concept by assessing clearance of HR HPVs and re-infections in in longitudinally followed WLWH with and without TB.

Thus, we have characterized Moscow cohort of women living with HIV-1 with clinical manifestations of TB as a unique population with a complex socio-behavioral profile which translates into a unique pattern of infection with high risk HPV types. Absence of the knowledge of HIV and TB status, and refusal to be ART-treated for those knowing their HIV+ status, along with drug use, smoking and other potentially harmful behaviors, indicated the lack of attention of these women to their health. At the same time, our data speak for the importance of timely provision to ART/TB-treatment and adherence to treatment regimen in this unique category of the population allowing to improve the health and quality of life of this vulnerable group of population. As a backlash, this may lead to enhanced infections with HR HPVs other than HPV 16, which emphasizes the need for efficient measures ensuring durable prevention of HR HPV infections, cervical abnormalities, and cancer. Standard treatment of HSIL to prevent progression to cancer in the settings of HIV infection is hard to accomplish: in our cohort, only 10/58 (17%) agreed to undergo examination of the cervix to detect eventual morphological abnormalities. Different approaches are needed to overcome health ignorance prevailing in this group of population.

Significant progress was lately achieved in the development of treatment(s) of HR HPV-associated precancerous lesions and cancer using specific immunotherapy/therapeutic HPV vaccines [108,109]. Decreased natural anti-HPV response required to preserve HPV-free status and oncologic remission, decreased tolerance of chemotherapy and radiation used to treat cervical cancer, and increased prevalence of anemia characteristic to WLWH [110], turn vaccination against HR HPV, primarily HPV 16, into potentially effective therapeutic instrument to preserve health of WLWH. An effective early measure could be a catch-up prophylactic HPV vaccination recently shown to significantly decrease positivity rates for DNA of HPV genotypes 16, 18, and 31, and for mRNA of HPV31 in women who tested positive for DNA of HPV 16, 18, and 31 prior to vaccination [110]. An alternative or a complement could be a therapeutic HPV vaccination targeting viral oncoproteins. The majority of therapeutic HPV vaccine candidates are based on E6 and E7 of HR HPVs indispensable for viral malignancy [108,111]. Several key pieces of information are needed to configure such treatment for WLWH, namely: knowledge of the distribution of HPV genotypes circulating in this group of population, E6 and E7 sequence variation, as well as a set of HPV-related biomarkers to define population(s) benefiting from such vaccination and optimal vaccination timing. Our study demonstrated that the prevalent HR HPV strain circulating among WLWH with TB is HPV 16 and that the sequences of its main oncoproteins in the strains circulating among WLWH with TB do not differ from those of HPV 16 circulating in other population groups, also outside of Russia. Altogether, this indicates that therapeutic HPV vaccines for this highly vulnerable population group could be the same as those developed for the general population, simplifying introduction of therapeutic HR HPV vaccination. Further studies are needed to define the optimal settings for their application.

## 5. Conclusions

WLWH with clinical TB manifestations are characterized by the high prevalence of infection with HPV 16 and other HR-HPV genotypes. We found that infections with HR HPVs did not depend on the form of TB infection (pulmonary versus extrapulmonary forms), but noted frequent infections with HR HPVs other than HPV 16 among WLWH with generalized TB, and/or low CD4+ T cell counts; specifically, those receiving combined ART/TB therapy, and in general, in WLWH on ART irrespective of their TB status. Prevalence in WLWH with TB of HR HPV types other than HPV 16 was not associated with their CD4+ T cell counts, duration of life with HIV, route of HIV infection, or risk behaviors, but depended on their sociodemographic characteristics, specifically, number of pregnancies reflecting sexual activity, and capacity to work, jointly reflecting their physical and emotional health. Associations between sociodemographic and clinical factors will be further delineated by multivariate analysis performed on a larger cohort of WLWH with and without TB. High prevalence in this cohort of HPV 16 strains close to those circulating in other population groups outside of Russia, specifically in the structure of E6/E7 oncoproteins, speaks in favor of the development and application of a universal therapeutic vaccine against HPV 16 as an additional therapeutic measure to prevent HPV-related cancers and preserve health of people living with HIV-1 and TB.

## Figures and Tables

**Table 1:** Demographic and clinical characteristics of women living with HIV-1 infected with *M. tuberculosis* (TB) (*n* = 58) when entering HPV study.

Parameter	n (%)	
**Age (** **full years)**		
21–29	9 (15.5)	
**30–39**	**34 (58.6)**	<0.05 ^a^
40–49	14 (24.1)	
≥ 60	1 (1.7%)	
**CD4 T cell counts (cells/mm^3^)**		
≥399	11 (18.9)	
200–399	15 (25.9)	
≤ 200	12 (20.7)	
≤ 67	20 (34.5)	
**Antiretroviral treatment (ART)**		
Untreated	21 (36.2)	
Treated (≤1 month)	6 (10.3)	
Treated (>1 month) including	31 (53.4)	
2 NRT + 1 NNRT	20 (64.5)	0.022 ^b^
2 NRT + 1(2) PI	11 (35.5)	
**TB forms**		
Group 1, TB with severe clinical manifestations (n = 10)	**10 (17.2)**	
Group 2 with other TB forms (n = 48)	**48 (82.8)**	<0.0001 ^c^
**Anti-TB therapy**		
Not receiving TB therapy	44 (75.9)	<0.0001^c^
Receiving TB therapy	14 (24.1)	
of Group 1, TB with severe clinical manifestations (n = 10)	5 (50)	0.036 ^d^
of Group 2 with other TB forms (n = 48)	9 (18.8)	
**Route of HIV transmission**		
Sexual contacts	**46 (79.3)**	<0.0001 ^c^
Other routes	**12 (20.7)**	
Intravenous drug use	10 (17.2)	
Dentistry	1 (1.7)	
Unknown	1 (1.7)	
**Merital status**		
Married	**7 (12.1)**	
Not married	**51 (87.9)**	<0.00001 ^c^
of not married, widowed	2 (3.4)	
of not married, divorced	8 (13.8)	
**Pregnancies (number)**		
Not reported	1 (1.72)	
0	10 (17.2)	
1	17 (29.3)	
≥2 including	**30 (51.8)**	<0.05 ^a^
2 pregancies	7 (12.1)	
3–4 pregancies	14 (24.1)	
5–7 pregancies	9 (15.5)	
**Abortions (number)**		
0	29 (50)	
1–2	20 (34.5)	
≥3	9 (15.5)	
3–4 abortions	7 (12.1)	
6 abortions	2 (3.4)	
**Sexual partners during last 6 month**		
0	8 (13.8)	
1	**45 (77.6)**	<0.0001 ^a^
≥2	5 (8.6)	
**Education**		
Secondary or secondary special^c^	**50 (86.2)**	<0.0001 ^c^
High school completed or incompleted	**8 (13.8)**	
**Occupation**		
Working	**19 (32.8)**	
Not working, including	**39 (67.2)**	0.0067 ^c^
Social subsidence	6 (10.3)	
Occasional work and support by relatives	33 (56.9)	
**Habits**		
No unhealthy habbits	**21 (36.2)**	
Unhealthy habits, including	**37 (63.8)**	0.003 ^c^
Smoking (>5 cigarettes/day)	32 (55.2)	
Intravenous drug use	15 (44.1)	
Regular use of alcohol	7 (12.1)	
Several unhealthy habits	16 (27.6)	

^a^ Group marked in bold is significantly larger than each of the other groups responding to the question. ^b^ Majority of WLWH and TB receiving ART were treated with a combination of 2 NRTI+1 NNRT. ^c^ Pair-wise comparison between two groups marked in bold (difference test, *p* < 0.05). ^d^ Percent receiving TB therapy in Group 1 with severe clinical manifestations was higher than % receiving TB therapy among patients in Group 2 with other TB forms.

**Table 2:** Characteristics of women living with HIV-1 co-infected with *M. tuberculosis* correlating with HR HPV infection.

				HR HPV-Positive			HPV 16 Positive		Positive for Non-HPV 16 HR HPVs			Positive for >=2 HR HPVs				HR HPV Negative	
Parameter	Groups	nn	nn	%	*p* Value	nn	%	*p* Value	nn	%	*p* Value	nn	%	*p* Value	nn	%	*p* Value
Age	<29 years	8	7	87.5	*0.053 (> “>39 years”)	**6**	**75**	*0.05 (> “>39 years”)	3	37.5	ns	2	25	ns	1	12.5	*0.05 (< “>39 years”)
					*0,1 (> “29–30 years”)			*0.02 (> “29–30 years”)									*0.1 (< “29–30 years”)
	29–39 years	34	20	57.1	ns	11	31.4		14	40		7	20		15	42.8	ns
	>39 years	16	7	46.6	ns	5	33		4	26.6		3	20		8	53.3	ns
CD4+ counts	<350	41	26	63,4	ns	18	43.9	ns	15	36.6	ns	10	24.4	ns	15	36.6	ns
	>350	17	8	47		4	23.5	ns	6	35.3		2	11.8		9	52.9	
	<200	32	22	68.8		16	50	*0.059 (> “>350 cells/mcl”)	12	37.5		8	25		11	31.4	
TB severity	Severe (generalized, fibrous cavernous) TB	10	7	70	ns	3	30	ns	6	60	0.08	3	30	ns	3	30	ns
	Other TB forms	48	27	56.2		19	39.6		15	31.3		9	18.8		20	41.7	
TB treatment	Yes	14	9	64.3	ns	3	21.4	ns	**9**	**64.3**	**0.012**	**6**	**42.9**	**0.018**	5	35.7	ns
	No	44	25	56.8		19	43.2		12	27.3		6	13.6		19	43.2	
ART (> 1 month)	Yes	23	15	65.2	ns	8	34.8	ns	**12**	**52.1**	**0.02**	7	30.4	ns	6	26.1	ns
	No	35	18	51.4		14	40		8	22.9		5	14.3		16	45.7	
Sexual partners	Yes	50	30	60		18	36		20	40		11	22		20	40	
	No	8	4	50		4	50		1	12.5		1	12.5		4	50	
Pregnancies	>=2*	31	**22**	**70.1**	**0.048**	14	45.1	ns	14	45.1	ns	8	25.8	ns	**9**	**29**	**0.041**
	<2	27	12	44.4		8	29.6		7	25.9		4	14.8		15	55.5	
Education	School	20	12	60	ns	9	45	ns	7	35	ns	5	25	ns	8	40	ns
	Professional, high school completed or not**	38	22	57.9		13	34.2		14	36.8		7	18,4		16	42.1	
Employment	Yes	18	12	66.6	ns	7	38.9	ns	**10**	**55.6**	**0.039**	**7**	**38,9**	**0.02**	6	33.3	ns
	No	40	22	55		15	37.5		11	27.5		5	12.5		18	45	
Smoking***	Yes***	32	20	62.5	ns	14	43.8	ns	13	40.6	ns	8	25	ns	12	37.5	ns
	No	26	14	53.8		8	30.8		8	30.8		4	15.4		12	46.2	

Characteristics significantly associated with infection with HR HPVs are marked in bold on a gray background (*p* < 0.05), characteristics associated, but with a lower significance (*p* < 0.1) are marked in gray. Ns—the difference is not significant (*p* > 0.1). The statistical significance of the difference between the two proportions was calculated according to the *t*-test (Statistica 11). *—The majority (23/31; 74.2%) reported two or more pregnancies; **—The majority (33/38; 86.8%) had a secondary specialized or incomplete higher education, only 5/38 (13.2%) graduated from high school; ***-The majority (29/32; 90.6%) smoked more than five cigarettes a day.

## Data Availability

The data supporting the results is provided in the main and supplementary figures and tables, raw data including the anonymized questionnaires filled by the patients is available from the corresponding co-authors upon the request.

## Supplementary Materials

The following are available online at https://www.mdpi.com/article/10.3390/biomedicines9060683/s1, Figure S1: Phylogenetic trees for E5 (A) and E6/E7 (B) genes of HPV 16 strains isolated from HIV/TB-infected women (MT353690-MT353699, MT353701- MT353712). Tree branches with reliability level of 90% and higher are depicted in red. Figure S2: Prevalence of HR HPVs in TB- negative WLWH (n=50) in relation to the demographic and clinical parameters of the group. Correlation of HR HPV DNA detected in cervical smears with the duration of life with HIV-1, but not age or CD4+ T cell counts at the time of analysis (A); Distribution of HR HPVs in different age categories (B); Distribution of HR HPVs in relation to the immune status of WLWH reflected by CD4+ T cell counts by the time of sampling (C). *p* values are indicated in the figure panels, Kruskall Wallis test (Statistica Axa 11). Figure S3: Positivity for HR HPVs (A), HPV 16 (B), HR HPVs other than HPV 16 (C), number of infecting HR HPV types (D) and of infecting HR HPV types other than HPV 16 (E) in HIV/TB-infected women receiving NRTI+1(2) NNRTI, NRTI+1(2) PI, or untreated. Kruskall Wallis test. Table S1: Characteristics of the control group of women living with HIV-1 (WLWH) uninfected with M. tuberculosis (MTB) (*n* = 50) compared to the study group of WLWH with TB (*n* = 58). CD4+ counts of two patients in the study group by the time of analysis were not available. Table S2: Comparison of demographic and clinical parameters in the control group of WLWH without TB (*n* = 50) depending on the stage of HIV-1 infection. Stage is defined as 3 or 4 (includes 4A, 4B, 4C by the criteria of the Ministry of Health of the Russian Federation). Mann-Whitney U Test; marked tests are significant at *p* <.05000 (Statistica Axa 11). Table S3: Comparison of prevalence of HR HPVs and multiplicity of HR HPV infection in WLWH with TB naïve to treatment and receiving ART. Patients grouped as receiving NRTI + 1(2) NNRTI, or NRT I+ 1(2) PI. Mann-Whitney U Test (with continuity correction) by variable ART regimen. Marked tests are significant at *p* <.05000. Table S4: Comparison of demographic and clinical parameters in the control group of WLWH without TB (*n* = 50) depending ART treatment status. Group 1, WLWH naïve to treatment or treated 1 month or less (*n* = 5); Group 2 (*n* = 45), WLWH on ART. Mann-Whitney U Test; marked tests are significant at *p* < 05000.
