# Supplementary material for: Prevalence and Risk Factors of Infection with High Risk Human Papilloma Viruses among HIV-Positive Women with Clinical Manifestations of Tuberculosis in a Middle-Income Country
Source: Biomedicines. 2021 Jun 16;9(6):683. doi: 10.3390/biomedicines9060683 (PMC8234035; doi:10.3390/biomedicines9060683)
Supplement: Supplementary file 1 [file biomedicines-09-00683-s001.zip › biomedicines-1183053-supplementary.pdf]

SUPPLEMENTARY DATA

Supplementary Table 1. Characteristics of the control group of women living with HIV-1 (WLWH) uninfected with *M. tuberculosis* (MTB) (n=50) compared to the study group of WLWH with TB (n=58). CD4+ counts of two patients in the study group by the time of analysis were not available.

Supplementary Table 2. Comparison of demographic and clinical parameters in the control group of WLWH without TB (n=50) depending on the stage of HIV-1 infection. Stage is defined as 3 or 4 (includes 4A, 4B, 4C by the criteria of the Ministry of Health of the Russian Federation). Mann-Whitney U Test; marked tests are significant at p <.05000 (Statistica Axa 11).

Supplementary Table 3. Comparison of prevalence of HR HPVs and multiplicity of HR HPV infection in WLWH with TB naïve to treatment, and receiving ART. Patients grouped as receiving NRTI + 1(2) NNRTI, or NRT I+ 1(2) PI. Mann-Whitney U Test (with continuity correction) by variable ART regimen. Marked tests are significant at p <.05000

Supplementary Table 4. Comparison of demographic and clinical parameters in the control group of WLWH without TB (n=50) depending ART treatment status. Group 1, WLWH naïve to treatment or treated 1 month or less (n=5); Group 2 (n=45), WLWH on ART. Mann-Whitney U Test; marked tests are significant at p <.05000

Supplementary Figure 1. Phylogenetic trees for E5 (A) and E6/E7 (B) genes of HPV 16 strains isolated from HIV/TB-infected women (MT353690-MT353699, MT353701- MT353712). Tree branches with reliability level of 90% and higher are depicted in red.

Supplementary Figure 2. Prevalence of HR HPVs in TB- negative WLWH (n=50) in relation to the demographic and clinical parameters of the group. Correlation of HR HPV DNA detected in cervical smears with the duration of life with HIV-1, but not age or CD4+ T cell counts at the time of analysis (A); Distribution of HR HPVs in different age categories (B); Distribution of HR HPVs in

relation to the immune status of WLWH reflected by CD4+ T cell counts by the time of sampling (C). p values are indicated in the figure panels, Kruskall Wallis test (Statistica Axa 11).

Supplementary Fig. 3. Positivity for HR HPVs (A), HPV 16 (B), HR HPVs other than HPV 16 (C), number of infecting HR HPV types (D) and of infecting HR HPV types other than HPV 16 (E) in HIV/TB-infected women receiving NRTI+1(2) NNRTI, NRTI+1(2) PI, or untreated. Kruskall Wallis test.

SUPPLEMENTARY TABLES

Supplementary Table 1. Characteristics of the control group of women living with HIV-1 (WLWH) without clinical manifestations of TB (n=50) compared to the study group of WLWH with TB (n=58). CD4+ counts of two patients in the study group by the time of analysis were not recorded. *, significant in difference test; ns, not significant (Statistica 11).

**Parameter**	**Control group, n (%)**	**Study group, n (%)**	**Statistical significance of difference**
**Age (full years)**			
21-29	5 (10)	9 (15,5)	ns
30-39	33 (66)	34 (58,6)	ns
40-49	10 (20)	14 (24,1)	ns
≥ 60	2 (4)	1 (1,7)	ns
**CD4 T cell counts (cells/mm^3)**			
≥ 399	28 (56)	11 (18,9)*	p=0,0001
200-399	9 (18)	15 (25,9)	ns
≤ 200	8 (16)	12 (20,7)	ns
≤ 67	5 (10)	20 (34,5)*	p=0,0026
**Antiretroviral treatment (ART)**			
untreated	2 (4)	21 (36,2)*	p=0,0002
treated (≤1 month)	3 (6)	6 (10,3)	ns
treated (>1 month)	45 (90)	31 (53,4)*	p=0,0001
treatment duration in those treated >1 month (years)	2.1±3.0	2.7±2.2	ns

Supplementary Table 2. Comparison of demographic and clinical parameters in the control group of WLWH without TB (n=50) depending on the stage of HIV-1 infection. Stage is defined as 3 or 4 (includes 4A, 4B, 4C by the criteria of the Ministry of Health of the Russian Federation). Mann-Whitney U Test; marked tests are significant at p <.05000 (Statistica Axa 11).

Parameter	Rank Sum (Stage 3)	Rank Sum (Stage 4)	U	Z	p-value	Z (adjusted)	p-value	Valid N (Stage 3)	Valid N (Stage 4)	2*1sided (exact p)
Age	414.0000	861.0000	183.0000	-2.37839	0.017389	-2.38486	0.017086	21	29	0.016412
Clinical data										
Years with HIV	505.0000	770.0000	274.0000	-0.58968	0.555404	-0.59389	0.552586	21	29	0.558708
CD4 at sampling	536.0000	640.0000	205.0000	1.47576	0.140010	1.47576	0.140010	19	29	0.141133
CD4 nadir	595.5000	580.5000	145.5000	2.73015	0.006331	2.73030	0.006328	19	29	0.005329
ART at sampling	517.0000	758.0000	286.0000	-0.35381	0.723482	-0.85997	0.389807	21	29	0.725876
ART duration, years	370.5000	805.5000	180.5000	-1.99227	0.046342	-1.99487	0.046058	19	29	0.044436
Presence in cervical smear of HPV DNA										
HPV16	517.5000	757.5000	286.5000	-0.34398	0.730861	-0.42566	0.670359	21	29	0.725876
HPV18	579.0000	696.0000	261.0000	0.84521	0.397993	2.05437	0.039941	21	29	0.401409
HPV31	529.0000	746.0000	298.0000	-0.11794	0.906118	-0.28666	0.774376	21	29	0.906993
HPV33	526.5000	748.5000	295.5000	-0.16708	0.867310	-0.26307	0.792498	21	29	0.860877
HPV35	529.0000	746.0000	298.0000	-0.11794	0.906118	-0.28666	0.774376	21	29	0.906993
HPV39	535.5000	739.5000	304.5000	0.00983	0.992158			21	29	
HPV45	514.5000	760.5000	283.5000	-0.40295	0.686986	-1.18696	0.235243	21	29	0.682459
HPV52	529.0000	746.0000	298.0000	-0.11794	0.906118	-0.28666	0.774376	21	29	0.906993
HPV56	591.0000	684.0000	249.0000	1.08108	0.279660	1.62431	0.104311	21	29	0.282466
HPV58	491.0000	784.0000	260.0000	-0.86487	0.387112	-1.29945	0.193791	21	29	0.390500
HPV59	514.5000	760.5000	283.5000	-0.40295	0.686986	-1.18696	0.235243	21	29	0.682459
HPV66	535.5000	739.5000	304.5000	0.00983	0.992158			21	29	
HR HPV (-)	545.0000	730.0000	295.0000	0.17690	0.859583	0.24651	0.805288	21	29	0.860877
HR HPV (+)	526.0000	749.0000	295.0000	-0.17690	0.859583	-0.24651	0.805288	21	29	0.860877
HR HPV other than HPV16 (+)	515.5000	759.5000	284.5000	-0.38329	0.701502	-0.55313	0.580178	21	29	0.696825
HPV16 and other (+)	526.5000	748.5000	295.5000	-0.16708	0.867310	-0.26307	0.792498	21	29	0.860877
>1 HR HPV	534.5000	740.5000	303.5000	-0.00983	0.992158	-0.01328	0.989402	21	29	0.984465
>2 HR HPVs	522.5000	752.5000	291.5000	-0.24570	0.805914	-0.43644	0.662516	21	29	0.800131
Number of infecting HR HPVs	523.5000	751.5000	292.5000	-0.22604	0.821167	-0.24819	0.803985	21	29	0.815222
Number of multiple HR HPV infections including HPV16	537.5000	737.5000	302.5000	0.02948	0.976479	0.03198	0.974489	21	29	0.968936

Supplementary Table 3. Comparison of prevalence of HR HPVs and multiplicity of HR HPV infection in WLWH with TB naïve to treatment, and receiving ART. Patients grouped as receiving NRTI + 1(2) NNRTI, or NRT I+ 1(2) PI. Mann-Whitney U Test (with continuity correction) by variable ART regimen. Marked tests are significant at p <.05000

**Treatment naïve (n=27) versus treated with NRTI+1(2)NNRTI (n=20)**										
variable	Rank Sum (treatment naive)	Rank Sum (NRTI+NNRTI)	U	Z	p-value	Z (adjusted)	p-value	Valid N (treatment naive)	Valid N (NRTI+NNRTI)	2*1sided (exact p)
HR HPV positivity (Y=1/N=0)	639.50	488.50	261.50	-0.17	0.86	-0.20	0.84	27.00	20.00	0.86
Positivity for HPV 16	677.00	451.00	241.00	0.61	0.54	0.74	0.46	27.00	20.00	0.54
nn HR HPVs	595.50	532.50	217.50	-1.12	0.26	-1.22	0.22	27.00	20.00	0.26
Positivity for HR HPVs other than HPV 16 (Y=1/N=0)	553.00	575.00	175.00	-2.03	0.04	-2.57	0.01	27.00	20.00	0.04
Summ of HR HPVs other than HPV 16	547.00	581.00	169.00	-2.16	0.03	-2.70	0.01	27.00	20.00	0.03
>=2 HR HPVs (Y=1/N=0)	563.50	564.50	185.50	-1.81	0.07	-2.78	0.01	27.00	20.00	0.07
**Treatment naïve (n=27) versus treated with NRTI+1(2)PI (n=11)**										
variable	Rank Sum (treatment naive)	Rank Sum (NRTI+PI)	U	Z	p-value	Z (adjusted)	p-value	Valid N (treatment naive)	Valid N (NRTI+PI)	2*1sided (exact p)
HR HPV positivity (Y=1/N=0)	482.00	259.00	104.00	-1.42	0.16	-1.67	0.09	27.00	11.00	0.16
Positivity for HPV 16	519.50	221.50	141.50	-0.21	0.83	-0.24	0.81	27.00	11.00	0.82
nn HR HPVs	458.50	282.50	80.50	-2.17	0.03	-2.38	0.02	27.00	11.00	0.03
Positivity for HR HPVs other than HPV 16 (Y=1/N=0)	454.00	287.00	76.00	-2.32	0.02	-2.95	0.00	27.00	11.00	0.02
Summ of HR HPVs other than HPV 16	452.00	289.00	74.00	-2.38	0.02	-3.02	0.00	27.00	11.00	0.02
>=2 HR HPV (Y=1/N=0)	478.00	263.00	100.00	-1.54	0.12	-2.64	0.01	27.00	11.00	0.12
**Treatment with NRTI+1(2)NNRTI (n=20) versus treated with NRTI+1(2)PI (n=11)**										
variable	Rank Sum (NRTI+NNRTI)	Rank Sum (NRTI+PI)	U	Z	p-value	Z (adjusted)	p-value	Valid N (NRTI+NNRTI)	Valid N (NRTI+PI)	2*1sided (exact p)
HR HPV positivity (Y=1/N=0)	290.50	205.50	80.50	-1.20	0.23	-1.44	0.15	20.00	11.00	0.23
Positivity for HPV 16	303.00	193.00	93.00	-0.68	0.50	-0.82	0.41	20.00	11.00	0.50
nn HR HPVs	302.00	194.00	92.00	-0.72	0.47	-0.76	0.45	20.00	11.00	0.48
Positivity for HR HPVs other than HPV 16 (Y=1/N=0)	305.00	191.00	95.00	-0.60	0.55	-0.69	0.49	20.00	11.00	0.56
Summ of HR HPVs other than HPV 16	310.50	185.50	100.50	-0.37	0.71	-0.41	0.68	20.00	11.00	0.70
>=2 HR HPV (Y=1/N=0)	318.50	177.50	108.50	-0.04	0.97	-0.05	0.96	20.00	11.00	0.95

Supplementary Table 4. Comparison of demographic and clinical parameters in the control group of WLWH without TB (n=50) depending ART treatment status. Group 1, WLWH naïve to treatment or treated 1 month or less (n=5); Group 2 (n=45), WLWH on ART. Mann-Whitney U Test; marked tests are significant at p <.05000.

Variable	Rank Sum (Group 1)	Rank Sum (Group 2)	U	Z	p-value	Z (adjusted)	p-value	Valid N (Group 1)	Valid N (Group 2)	2*1sided (exact p)
Age	134.0000	1141.000	106.0000	0.19403	0.846154	0.19456	0.845740	5	45	0.850839
Clinical data										
Stage 3 vs Stage 4	105.0000	1170.000	90.0000	-0.71144	0.476813	-0.83205	0.405380	5	45	0.488327
Years with HIV	126.5000	1148.500	111.5000	-0.01617	0.987100	-0.01628	0.987008	5	45	0.975008
CD4 status at sampling	93.5000	1082.500	47.5000	0.83054	0.406234	0.94521	0.344555	3	45	0.412697
CD4 nadir	40.0000	1136.000	34.0000	-1.40553	0.159865	-1.40560	0.159842	3	45	0.168941
ART duration, years	51.0000	1125.000	45.0000	-0.93702	0.348750	-0.93824	0.348121	3	45	0.365865
Presence in cervical smear of HPV DNA										
HPV16	162.5000	1112.500	77.5000	1.11566	0.264567	1.38056	0.167414	5	45	0.266694
HPV18	120.0000	1155.000	105.0000	-0.22637	0.820916	-0.55021	0.582179	5	45	0.826320
HPV31	120.0000	1155.000	105.0000	-0.22637	0.820916	-0.55021	0.582179	5	45	0.826320
HPV33	157.5000	1117.500	82.5000	0.95397	0.340098	1.50207	0.133081	5	45	0.342661
HPV35	120.0000	1155.000	105.0000	-0.22637	0.820916	-0.55021	0.582179	5	45	0.826320
HPV39	127.5000	1147.500	112.5000	0.01617	0.987100			5	45	
HPV45	122.5000	1152.500	107.5000	-0.14552	0.884299	-0.42866	0.668170	5	45	0.875492
HPV52	120.0000	1155.000	105.0000	-0.22637	0.820916	-0.55021	0.582179	5	45	0.826320
HPV56	105.0000	1170.000	90.0000	-0.71144	0.476813	-1.06892	0.285105	5	45	0.488327
HPV58	130.0000	1145.000	110.0000	0.06468	0.948432	0.09717	0.922588	5	45	0.950039
HPV59	122.5000	1152.500	107.5000	-0.14552	0.884299	-0.42866	0.668170	5	45	0.875492
HPV66	127.5000	1147.500	112.5000	0.01617	0.987100			5	45	
Summ HR HPVs	139.0000	1136.000	101.0000	0.35572	0.722051	0.39057	0.696113	5	45	0.730016
Other HR HPVs (Y=1/N=0)	102.5000	1172.500	87.5000	-0.79228	0.428196	-1.14333	0.252901	5	45	0.430343
Count if >1	147.5000	1127.500	92.5000	0.63059	0.528307	0.85229	0.394052	5	45	0.529075
Count if 0	125.0000	1150.000	110.0000	-0.06468	0.948432	-0.09012	0.928189	5	45	0.950039
HPV16+another	108.0000	1167.000	93.0000	-0.61442	0.538936	-0.66640	0.505154	5	45	0.550041
HPV16+another (Y=1/N=0)	132.5000	1142.500	107.5000	0.14552	0.884299	0.22913	0.818769	5	45	0.875492
Count if >2	112.5000	1162.500	97.5000	-0.46890	0.639140	-0.83292	0.404891	5	45	0.637526
Any HR HPV	130.0000	1145.000	110.0000	0.06468	0.948432	0.09012	0.928189	5	45	0.950039

SUPPLEMENTARY FIGURES

Supplelementary Fig. 1. Phylogenetic trees for E5 (A) and E6/E7 (B) genes of HPV 16 strains isolated from HIV/TB-infected women (MT353690-MT353699, MT353701- MT353712). Tree branches with reliability level of 90% and higher are depicted in red.

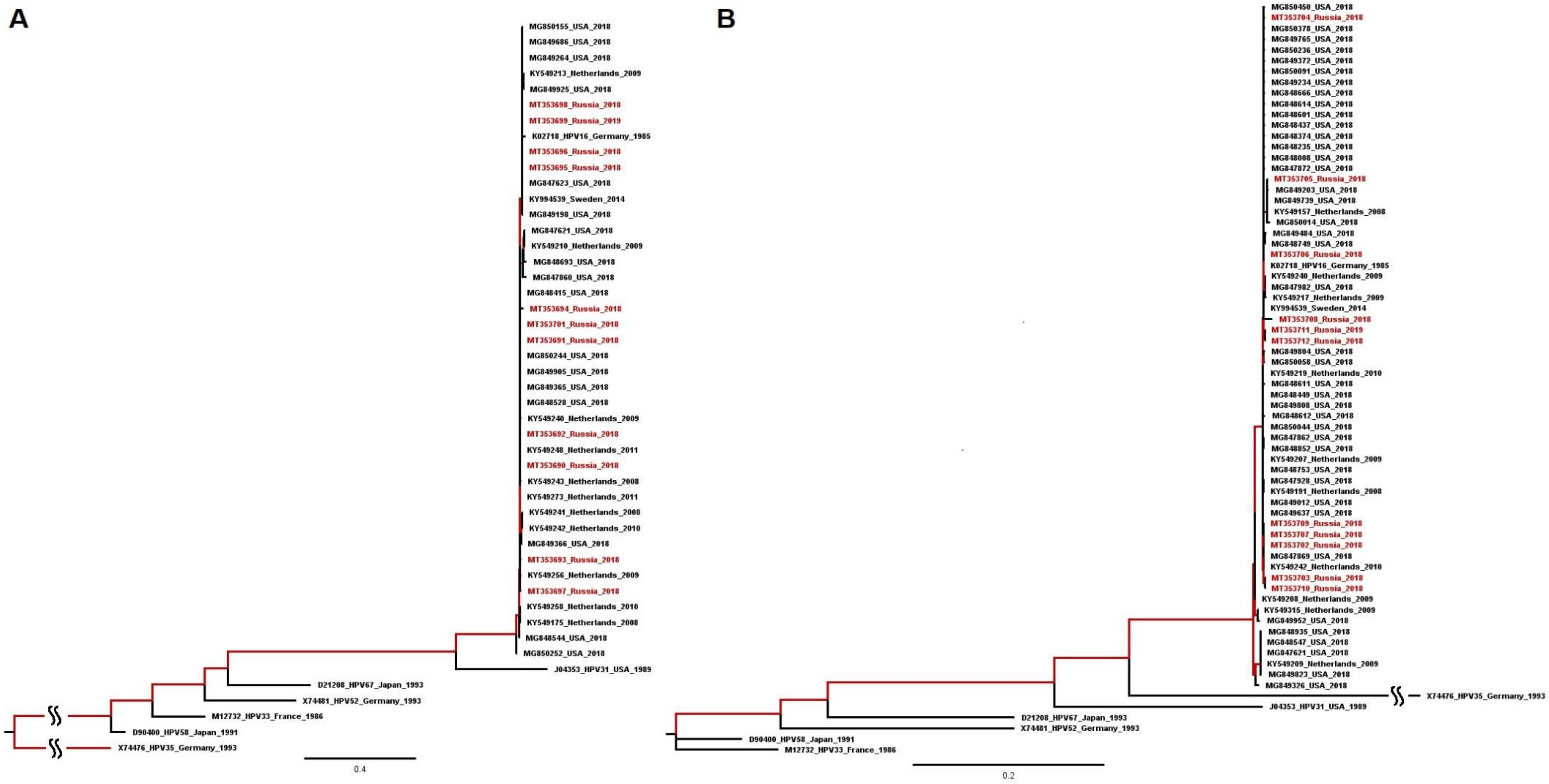

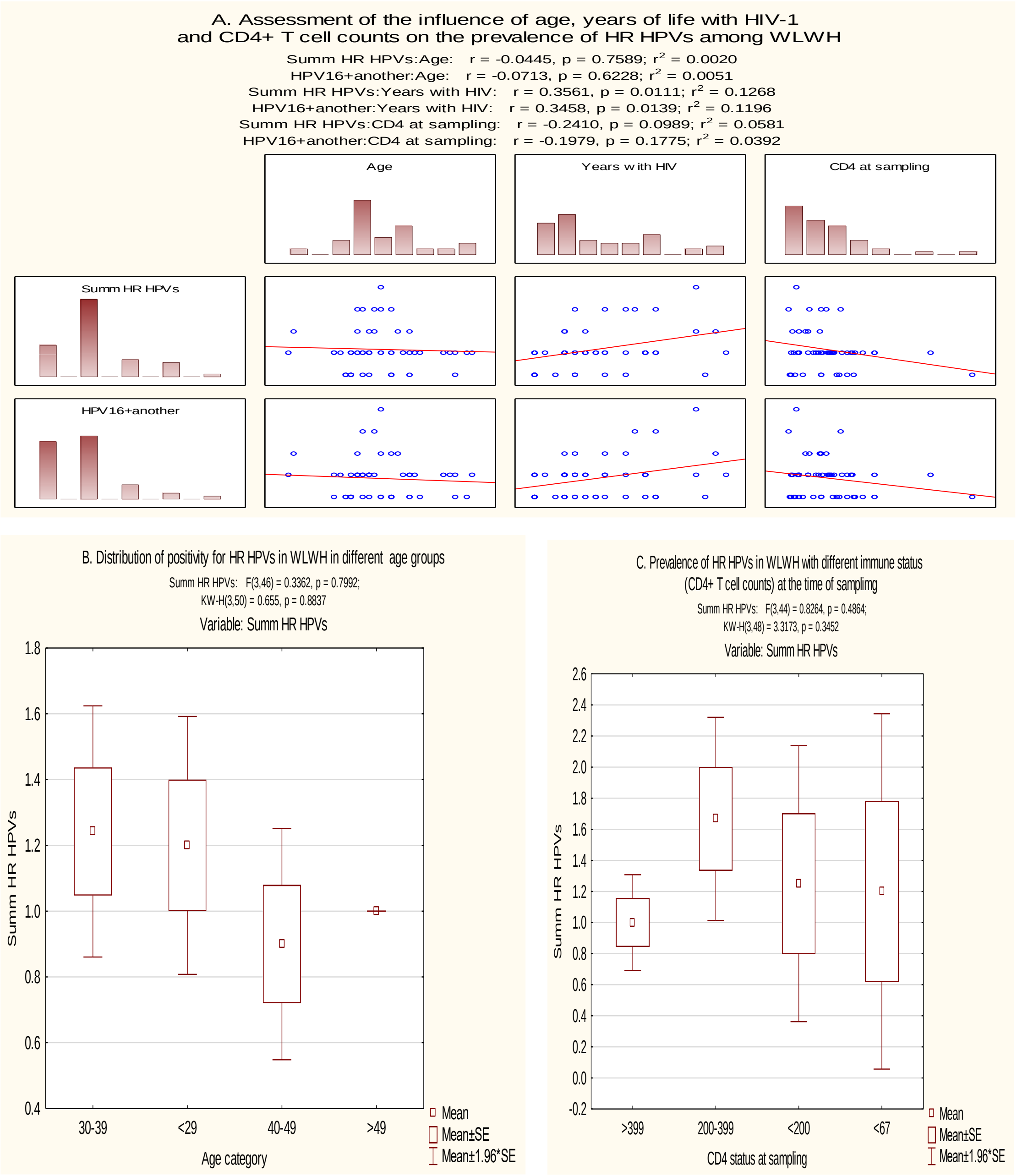

Supplementary Figure 2. Prevalence of HR HPVs in WLWH without TB (n=50) in relation to the demographic and clinical parameters of the group. Correlation of HR HPV DNA detected in cervical smears with the duration of life with HIV-1, but not age or CD4+ T cell counts at the time of analysis (A); Number of infecting HR HPV genotypes in different age categories (B); Number of infecting HR HPV genotypes in relation to the immune status of WLWH reflected by CD4+ T cell counts by the time of sampling (C). p values are indicated in the panels, Kruskall Wallis test (Statistica 11).

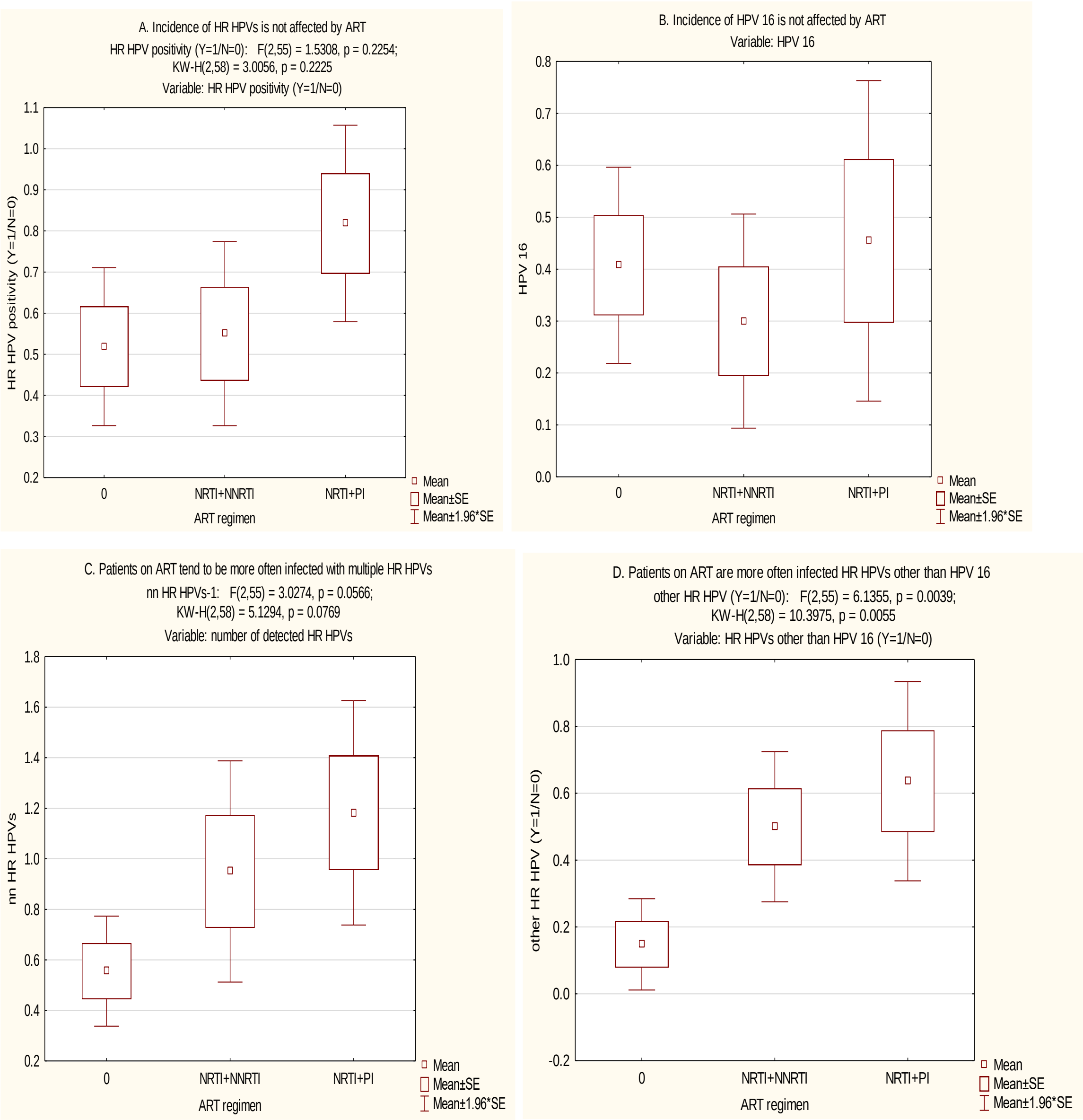

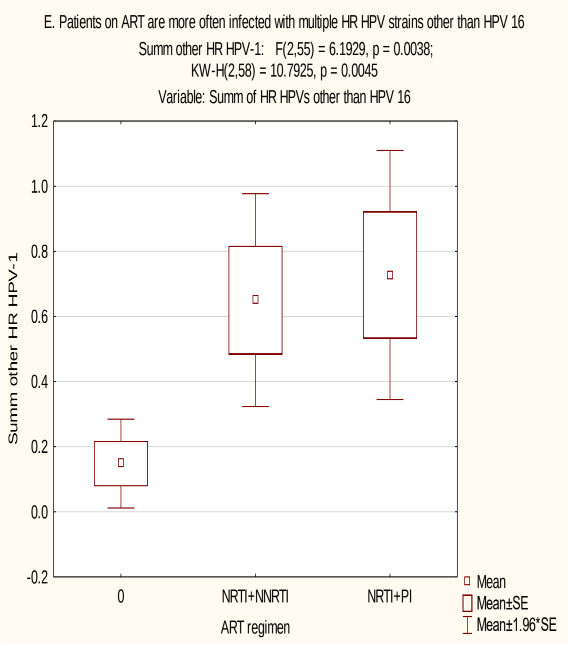

Supplementary Fig. 3. Positivity for HR HPVs (A), HPV 16 (B), HR HPVs other than HPV 16 (C), number of infecting HR HPV types (D) and of infecting HR HPV types other than HPV 16 (E) in HIV/TB-infected women receiving NRTI+1(2) NNRTI, NRTI+1(2) PI, or untreated. Kruskall Wallis test.
